# Ru‐Doping‐Engineered Oxygen Vacancies in MoO_3‐_ *_x_* Nanostructured Films With Ultrahigh Capacitance for Flexible Asymmetric Supercapacitors

**DOI:** 10.1002/advs.77131

**Published:** 2026-08-17

**Authors:** Xiaoqing Bin, Minhao Sheng, Norman C.‐R. Chen, Xiangyang Liu, Yingji Zhao, Yingyi Liao, Wenxiu Que, Yusuke Asakura, Yusuke Yamauchi

**Affiliations:** ^1^ Electronic Materials Research Laboratory, Key Laboratory of the Ministry of Education, International Center For Dielectric Research, Shaanxi Engineering Research Center of Advanced Energy Materials and Devices, School of Electronic Science and Engineering Xi'an Jiaotong University Xi'an P. R. China; ^2^ Department of Materials Process Engineering, Graduate School of Engineering Nagoya University Nagoya Japan; ^3^ Molecular Science and Technology Program, Taiwan International Graduate Program Academia Sinica Taipei Taiwan; ^4^ International Graduate Program of Molecular Science and Technology (NTU‐MST) National Taiwan University Taipei Taiwan; ^5^ Australian Institute for Bioengineering and Nanotechnology (AIBN) The University of Queensland Brisbane Queensland Australia; ^6^ Department of Convergent Biotechnology and Advanced Materials Science Kyung Hee University 1732 Deogyeong‐daero, Giheung‐gu, Gyeonggi‐do Yongin‐si South Korea

**Keywords:** defect engineering, electronic structure modulation, flexible supercapacitors, oxygen‐vacancy engineering, Ru‐doped MoO_3_ film

## Abstract

Molybdenum trioxide (MoO_3_) is a promising pseudocapacitive material owing to its high theoretical charge‐storage capacity, but its practical application is limited by low electrical conductivity and sluggish ion transport. Here, we report a synergistic defect‐engineering strategy that combines in situ Ru doping with simultaneous oxygen‐vacancy generation to synthesize Ru‐doped MoO_3‐*x*_ (Ru‐MoO_3‐*x*_)via a one‐step solvothermal reaction, followed by assembly into freestanding, flexible film electrode by vacuum filtration. Ru incorporation induces a dual nanobelt‐nanowire architecture and enriches oxygen vacancies as electrochemically active sites, enabling efficient redox reactions and charge transport. Density functional theory calculations and ultraviolet photoelectron spectroscopy reveal that Ru‐induced defect engineering narrows the bandgap, lowers the work function, and shifts the Fermi level upward, thereby enhancing intrinsic conductivity. The optimized Ru‐MoO_3‐_ *_x_* electrode delivers an ultrahigh specific capacitance of 2047.5 F g^−1^ at 1 A g^−1^, approaching the theoretical capacitance limit of MoO_3_ (∼2700 F g^−1^ at 1 V), with excellent cycling stability. Asymmetric supercapacitors assembled with Ru‐MoO_3‐_ *_x_* achieve a high energy density of 40.9 Wh kg^−1^ in acidic electrolyte and a wide operating voltage window of 2.4 V in organic ionic‐liquid electrolyte, while maintaining mechanical robustness under severe bending and powering commercial electronics. This work provides a general strategy for defect‐regulated, high‐performance flexible energy‐storage devices.

## Graphical abstract

Ru‐doping‐induced defect engineering enables freestanding Ru–MoO_3‐*x*_ flexible films with enriched oxygen vacancies, modulated band structure, and enhanced charge transport. The optimized electrode delivers ultrahigh capacitance approaching the theoretical limit of MoO_3_, while assembled asymmetric supercapacitors exhibit high energy density, wide‐voltage operation, and robust mechanical flexibility for practical energy‐storage applications.

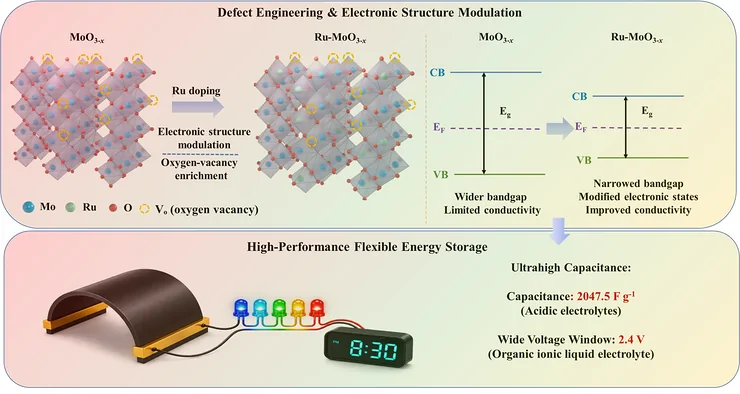

## 1 Introduction

Supercapacitors, also referred to as electrochemical capacitors, have attracted significant attention as advanced energy storage devices that bridge the performance gap between conventional capacitors and batteries. Their exceptional power density, rapid charge‐discharge capability, long cycle life, and excellent thermal stability render them highly promising for applications that demand both high energy and power densities [1, 2, 3, 4]. Unlike traditional batteries, which store energy via bulk chemical reactions, supercapacitors store energy through two principal mechanisms: electrical double‐layer capacitance (EDLC) and pseudocapacitance. EDLC arises from electrostatic charge accumulation at the electrode‐electrolyte interface and EDLC electrodes typically employ carbon‐based materials with high surface area, conductivity, and robust cyclability, such as activated carbon, carbon nanotubes, and graphene [5, 6, 7]. However, carbon‐based materials typically exhibit limited specific capacitance (100–300 F g^−1^), as charge storage is confined to the electrical double‐layer mechanism [7, 8]. In contrast, pseudocapacitance involves fast and reversible Faradaic reactions occurring at or near the electrode surface, enabling substantially higher specific capacitance through rapid redox processes in materials such as transition metal oxides and conducting polymers (RuO_2_, MnO_2_, PPy, PANI, etc.), which can undergo rapid surface redox transitions and thus store significantly more charge than purely capacitive carbon electrodes [9, 10, 11].

To achieve higher energy storage, significant attention has been devoted to pseudocapacitive materials, particularly transition metal oxides, due to their high theoretical specific capacitances and diverse redox behaviors. For instance, manganese dioxide (MnO_2_), ruthenium oxide (RuO_2_), cobalt oxide (Co_3_O_4_), iron oxide (Fe_2_O_3_), and molybdenum trioxide (MoO_3_) exhibit theoretical specific capacitances typically in the range of 1000–3000 F g^−1^, originating from their versatile multivalent redox reactions [2, 12, 13, 14]. Among them, MoO_3_ is regarded as a highly promising pseudocapacitive candidate owing to its low toxicity, low cost, natural abundance, and excellent electrochemical stability, particularly in its orthorhombic α‐phase, which features a unique layered structure composed of MoO_6_ octahedra stacked in stacked bilayers and held together by weak van der Waals interactions [12, 15, 16]. This layered architecture provides open galleries for rapid ion intercalation/deintercalation, enabling it to act as an “intercalation pseudocapacitor” that exhibits characteristics intermediate between batteries and capacitors by reversibly accommodating various cations (H^+^, Li^+^, Na^+^, etc.) between its sheets without drastic structural strain, thus facilitating fast pseudocapacitive charge storage throughout the bulk rather than being confined to the surface. Furthermore, the accessibility of multiple oxidation states of molybdenum (+3 to +6) enables rich redox activity and multi‐electron transfer processes (Mo^6+^ to Mo^5+^ to Mo^4+^), whereby each Mo center can contribute more than one electron, endowing MoO_3_ with an ultrahigh theoretical specific capacitance. This performance can be further enhanced by synthesizing MoO_3_ into various nanostructures, such as nanorods, nanosheets, and hierarchical architectures, which increase the density of electrochemically active sites and facilitate ion diffusion [15, 16, 17, 18, 19].

Although MoO_3_ exhibits several attractive features, its practical application is severely constrained by intrinsically low electrical conductivity and surface‐confined redox reactions—limitations common to pseudocapacitive materials that significantly restrict charge utilization [20, 21, 22]. As a wide‐bandgap n‐type semiconductor (∼3 eV), MoO_3_ exhibits low electrical conductivity typically in the range of 10^−6^–10^−4^ S cm^−1^ at room temperature, which is several orders of magnitude lower than that of carbon‐based or metallic conductors. Such poor conductivity leads to pronounced internal resistance (IR) drop and incomplete utilization of electroactive material at high charge–discharge rates, resulting in practical capacitance values far below the theoretical values. Moreover, inefficient electron percolation throughout the bulk during repeated cycling accelerates capacitance fading, further limiting the long‐term electrochemical performance of MoO_3_.

To overcome the intrinsic limitations of MoO_3_, particularly its poor electrical conductivity, numerous strategies have been developed, including composite design, elemental doping, nanoengineering, and defect chemistry [15, 23, 24, 25, 26]. Among these strategies, the introduction of oxygen vacancies in combination with elemental doping has emerged as a particularly effective approach for tailoring the electronic structure, thereby enhancing the intrinsic conductivity and electrochemical performance of MoO_3_ [23, 27, 28]. The formation of oxygen vacancies as point defects increases the density of adsorption and activation sites for redox reactions and can simultaneously introduce additional charge carriers, such as electrons or holes, thereby improving both the electrical conductivity and electrochemical performance [15, 29, 30, 31]. Accordingly, MoO_3‐_ *_x_* with well‐controlled oxygen vacancies exhibits near‐theoretical capacity, good rate capability, and substantially enhanced cycling stability [32]. Xiao et al. [17] synthesized oxygen‐vacancy‐rich MoO_3_ (MoO_3‐_ *_x_*) via hydrogenation treatment, yielding electrodes that exhibited a specific capacitance of 337 F g^−1^ in LiCl electrolyte, along with enhanced electrical conductivity and excellent rate performance. Kim et al. [28] developed a one‐step microwave‐assisted hydrothermal method to synthesize oxygen‐deficient MoO_3‐_ *_x_*, demonstrating that introducing oxygen vacancies into transition metal oxides significantly enhanced the charge‐storage kinetics of redox‐active materials. Li et al. [33] prepared MoO_3‐_ *_x_* via ethanol‐assisted reduction of molybdenum trioxide, achieving a high specific capacitance of 420 F g^−1^ together with excellent cycling stability.

Furthermore, metal doping has emerged as an effective approach for tailoring the electronic structure and enhancing the physicochemical properties of functional materials. Sun et al. [29] induced oxygen vacancies (OV) on the Ti_3_O_5_ surface via Fe doping, thereby enabling highly sensitive electronic transitions. Fe doping promoted the generation of free electrons by reducing the electronic bandgap, which lowers the energy barrier for valence‐band electrons to transition into the conduction band. Zhang et al. [26] reported that tungsten (W) doping enriched oxygen vacancies in MoO_3_ nanobelts, thereby further enhancing their ion‐storage performance. Xu et al. [23] demonstrated that Ce doping in the OV‐MoO_3_ lattice introduced additional oxygen vacancies. Leveraging the synergistic effects of these oxygen vacancies, high specific surface area, and accelerated ion diffusion and charge transport, the optimized OV‐MoO_3_/Ce electrode achieved high specific capacitances of 1439.0 F g^−1^ at 2 mV s^−1^ and 1446.3 F g^−1^ at 5 A g^−1^, along with excellent cycling stability. In addition, Ru doping has been reported to modulate the electronic structure and enhance electrical conductivity and charge‐transfer kinetics in transition‐metal oxides. These effects are often discussed in relation to the unique electronic characteristics of Ru [18, 34]. Li et al. [35] demonstrated that the synergistic interplay between Ru doping and tunable oxygen vacancies in TiO_2_ significantly improved its photocatalytic activity for CO_2_ methanation. Peng et al. [36] synthesized Ru‐doped Na_3_V_2_O_2_(PO_4_)_2_F hollow microspheres via a low‐temperature solvothermal approach. When evaluated as a cathode material in sodium‐ion batteries (SIBs), these microspheres demonstrated a high specific capacity of 102.5 mAh g^−1^ together with remarkable long‐term cycling stability. Collectively, studies suggest that the combination of oxygen‐vacancy engineering and Ru doping is an effective strategy for regulating the electronic structure and charge‐transport properties of transition metal oxides. Such insights provide a rational basis for designing MoO_3_‐based electrodes with enhanced electrochemical performance, particularly for capacitive charge‐storage applications.

Here, Ru was introduced while oxygen vacancies were generated in MoO_3_ via a one‐step solvothermal reaction, yielding a freestanding, binder‐free flexible film electrode material of ruthenium‐doped molybdenum trioxide with oxygen vacancies (Ru‐MoO_3‐_ *_x_*) that exhibits metallic luster. The optimized Ru‐MoO_3‐_ *_x_* flexible film electrode demonstrates an ultrahigh specific capacitance of 2047.5 F g^−1^ at 1 A g^−1^, approaching the theoretical limit of MoO_3_. The significant enhancement is primarily attributed to this synergistic dual‐defect engineering. The incorporation of Ru not only modulates the morphology and electronic structure of MoO_3‐_ *_x_* but also induces lattice distortion and promotes the formation of oxygen vacancies. These changes may facilitate intervalence charge transfer (IVCT) between mixed‐valence Mo species, thereby enhancing the intrinsic electrical conductivity and charge‐transport properties. Furthermore, when assembled into an asymmetric supercapacitor in 3 M H_2_SO_4_ as an electrolyte, the device achieves a high energy density of 40.9 Wh kg^−1^ and is capable of powering an LED lamp for up to 3 min and an alarm clock for over 10 min. As depicted in Figure 1, the atomic structure of Ru‐MoO_3‐_ *_x_* is constructed upon MoO_6_ octahedra forming a bilayer framework, in which oxygen‐vacancy‐related defects are present, and Ru is successfully introduced into the MoO_3‐_ *_x_*‐based film. The as‐prepared film not only displays a metallic luster and possesses excellent mechanical flexibility (effectively eliminating the “dead volume” typical of slurry‐cast electrodes), but also provides an ideal platform to decode the underlying charge storage mechanisms, bridging macroscopic performance with microscopic desolvation kinetics, thereby enabling its successful application in aqueous, flexible solid‐state, and organic supercapacitors.

**FIGURE 1:**
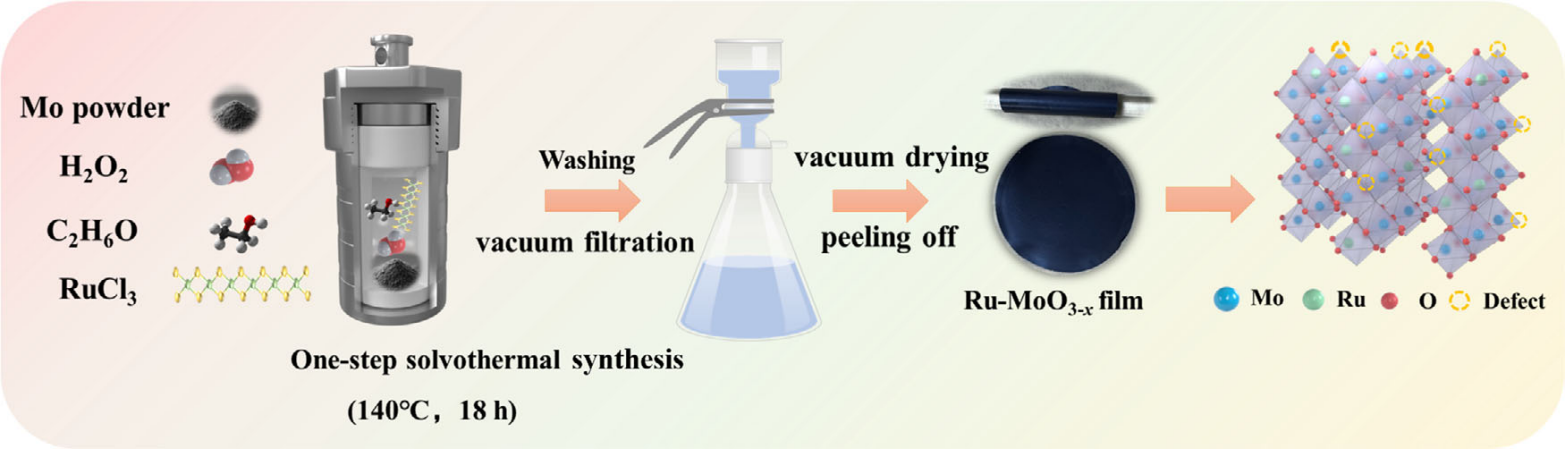
Schematic diagram of the preparation of a flexible ruthenium‐doped molybdenum trioxide with oxygen vacancies (Ru‐MoO_3‐_ *_x_*) film.

## 2 Results and Discussion

The oxygen‐vacancy‐rich molybdenum trioxide (MoO_3‐_ *_x_*) was synthesized both with and without Ru doping. By varying the amount of RuCl_3_ added to the precursor solution (1, 3, and 6 mg), the resulting samples are denoted as Ru‐MoO_3‐_ *_x_*‐1, Ru‐MoO_3‐_ *_x_*‐3, and Ru‐MoO_3‐_ *_x_*‐6, respectively. The surface morphologies of MoO_3‐_ *_x_* and Ru‐MoO_3‐_ *_x_* flexible films with different Ru‐doping levels were investigated using aberration‐corrected scanning transmission electron microscopy (AC‐STEM, JEM‐ARM200F). As depicted in Figure 2a–d, all samples display the characteristic nanobelt morphology of MoO_3_. The uniform nanobelt morphology of MoO_3‐_ *_x_*, and its elemental composition (Mo and O), are further confirmed by the STEM image and the corresponding elemental mapping in Figure S1. Notably, the Ru‐MoO_3‐_ *_x_*‐3 film exhibits both nanobelt and nanowire morphologies, which is consistent with the elemental distributions of Mo, O, and Ru elements (Figure 2g). This Ru‐doping‐induced dual morphology, consisting of nanobelts and nanowires, increases the density of electrochemically active sites, thereby further enhancing the capacitive performance of the material. The high‐resolution ADF‐STEM images (Figure 2e,f) were subjected to fast Fourier transform (FFT) analysis, revealing an identical interplanar spacing of 0.23 nm for both morphologies, which can be indexed to the (060) crystal plane. Moreover, high‐resolution annular dark‐field STEM (ADF‐STEM) imaging of Ru‐MoO_3‐_ *_x_*‐3 (Figure 2f) shows local variations in the intensity and periodicity of the cation columns, as highlighted by the yellow circles, suggesting the presence of local structural disorder.

**FIGURE 2:**
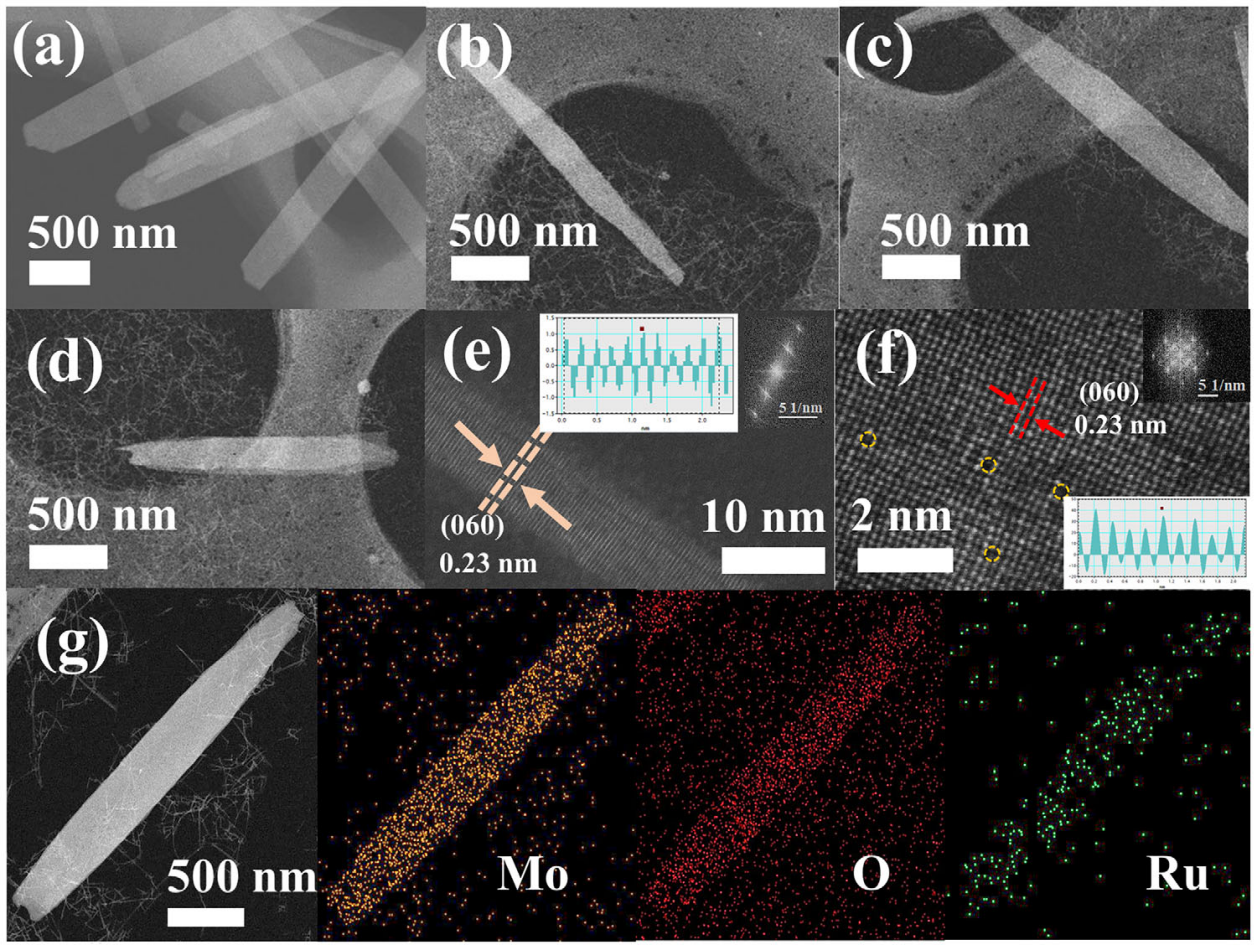
STEM images for the surfaces of MoO_3‐_ *_x_* and Ru‐MoO_3‐_ *_x_* flexible films with varying Ru concentrations: (a) MoO_3‐_ *_x_*, (b) Ru‐MoO_3‐_ *_x_*‐1, (c) Ru‐MoO_3‐_ *_x_*‐3, (d) Ru‐MoO_3‐_ *_x_* ‐6. Annular dark field‐scanning transmission electron microscopy (ADF‐STEM) images of the Ru‐MoO_3‐_ *_x_*‐3 film: (e) nanowires; (f) nanobelts, and (g) the corresponding elemental mapping.

Figure 3a presents the XRD patterns of MoO_3‐_ *_x_* and Ru‐doped MoO_3‐_ *_x_* flexible films with various Ru contents. All films exhibit the characteristic diffraction peaks of (020), (040), and (060) planes, which are indexed to orthorhombic α‐MoO_3_ (PDF#35‐0609) [20]. The XRD patterns of the Ru‐MoO_3‐_ *_x_*‐1, Ru‐MoO_3‐_ *_x_*‐3, and Ru‐MoO_3‐_ *_x_*‐6 films are nearly identical to that of pristine MoO_3‐_ *_x_*, with no detectable impurity peaks, indicating that Ru doping does not alter the primary crystal structure of MoO_3‐_ *_x_*. However, the (110) and (021) diffraction peaks in the Ru‐doped films are significantly weakened and nearly disappear compared to the undoped MoO_3‐_ *_x_*. This attenuation is likely associated with Ru‐induced lattice distortion in MoO_3_, which can influence the formation and concentration of oxygen vacancies. Raman spectroscopy provides complementary evidence for local structural disorder (Figure 3b). The characteristic vibrational modes of MoO_3‐_ *_x_* are observed, including a rigid‐chain translational mode at about 150 cm^−1^ (A_g_, B_1g_), the wagging modes of terminal oxygen atoms at 285 cm^−1^ (B_2g_, B_3g_), asymmetric stretching of the Mo‐O‐Mo bridge along the c‐axis at 666 cm^−1^ (B_2g_, B_3g_), symmetric stretching of terminal oxygen atoms at 820 cm^−1^ (A_g_, B_1g_), and the asymmetric stretching at 995 cm^−1^ (A_g_, B_1g_) [37, 38, 39, 40]. Notably, Ru‐doped MoO_3‐_ *_x_* exhibits an enhanced Raman‐active vibration at 850 cm^−1^, which may be attributed to the modification of the local electronic environment associated with Ru addition, leading to increased polarizability of the Mo─O─Mo vibration [40, 41]. Concurrently, the remaining characteristic peaks exhibit blue shifts, peak broadening, and reduced intensities, with some modes disappearing entirely. Collectively, these spectral changes are consistent with Ru‐associated lattice distortion and local structural disorder, while the increased oxygen‐vacancy concentration is further supported by the EPR and XPS results [42, 43].

**FIGURE 3:**
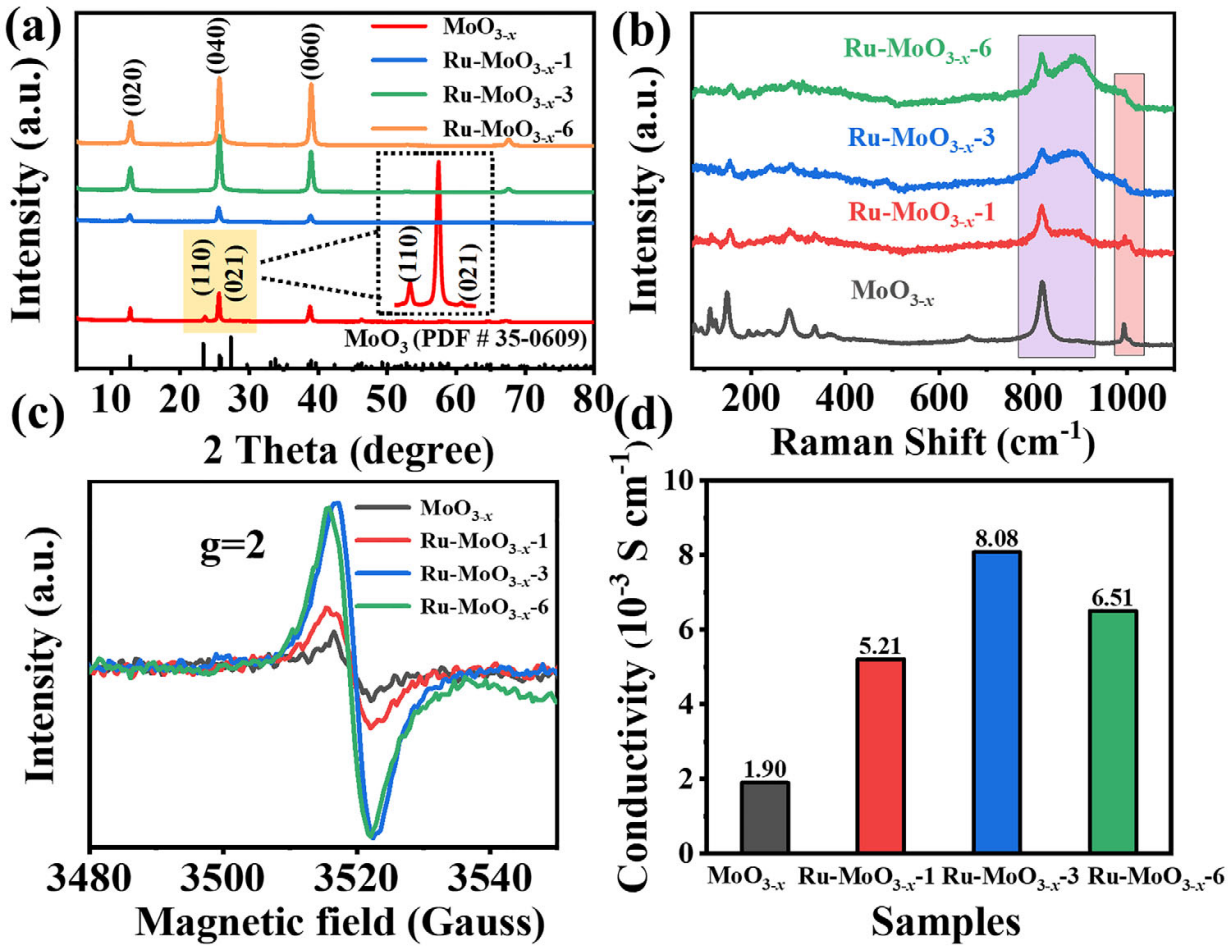
(a) XRD patterns, (b) Raman spectra, (c) Electron paramagnetic resonance (EPR) spectra, and (d) electrical conductivity of MoO_3‐_ *_x_* and Ru‐MoO_3‐_ *_x_* flexible films with varying Ru concentrations.

To gain deeper insights into the effects of Ru doping on the electronic properties and oxygen vacancy formation in MoO_3‐_ *_x_*, electron paramagnetic resonance (EPR) spectroscopy was conducted on flexible films of pristine MoO_3‐_ *_x_* and Ru‐doped MoO_3‐_ *_x_* (Ru‐MoO_3‐_ *_x_*) with varying Ru content. As a sensitive probe for unpaired electrons, EPR provides evidence for paramagnetic centers associated with oxygen‐vacancy formation. As shown in Figure 3c, the symmetric resonance signal centered at g = 2.0 is assigned to oxygen‐vacancy‐associated paramagnetic centers, including Mo^5+^ species formed through the localization of vacancy‐derived electrons. The EPR signal intensity of Ru‐MoO_3‐_ *_x_* is significantly stronger than that of the undoped MoO_3‐_ *_x_*, indicating a greater relative abundance of oxygen‐vacancy‐associated paramagnetic centers. Notably, the Ru‐MoO_3‐_ *_x_*‐3 sample exhibits the most intense EPR signal, suggesting the highest relative concentration of oxygen‐vacancy‐associated paramagnetic centers among the samples. Together with the above Raman analysis, these observations support the conclusion that Ru doping effectively promotes the formation of oxygen vacancies. To evaluate the effect of Ru doping on charge transport, electrical conductivity measurements were performed using the four‐probe method (Supporting Information, Equations S1 and S2, Table S1). As presented in Figure 3d, Ru doping dramatically enhances the electrical conductivity of MoO_3‐_ *_x_*. In particular, the Ru‐MoO_3‐_ *_x_*‐3 sample achieves a maximum conductivity of 8.08 × 10^−3^ S cm^−1^. The remarkable enhancement can be attributed to two synergistic effects: (i) oxygen vacancies can act as donor defects and increase the electron concentration, and (ii) Ru‐induced lattice distortion may facilitate charge transport across grain boundaries [44, 45]. Taken together, these results demonstrate that Ru doping effectively tunes the electronic structure of MoO_3‐_ *_x_* flexible films, significantly enhancing their electrical conductivity likely through the introduction of abundant oxygen vacancies and Ru‐induced lattice distortions.

The electronic structure and local coordination environment of MoO_3‐_ *_x_* and Ru‐MoO_3‐_ *_x_* were investigated using X‐ray absorption fine structure (XAFS) spectroscopy. As shown in the Mo K‐edge X‐ray absorption near‐edge structure (XANES) spectra (Figure 4a), the absorption edge of Ru‐MoO_3‐_ *_x_* shifts slightly toward lower energy relative to that of MoO_3‐_ *_x_*, while remaining at a higher energy than that of the metallic Mo reference. This negative shift indicates a decrease in the average oxidation state of Mo upon Ru doping, consistent with the increased formation of oxygen vacancies in Ru‐MoO_3‐_ *_x_*. The Fourier‐transformed Mo K‐edge extended X‐ray absorption fine structure (FT‐EXAFS) spectra (Figure 4b) show prominent peaks at approximately 1.5 and 2.9 Å, corresponding to Mo─O and Mo─Mo coordination shells, respectively. While the Mo‐O coordination features of Ru‐MoO_3‐_ *_x_* are similar to those in MoO_3‐_ *_x_*, the intensity of the Mo‐Mo peak of Ru‐MoO_3‐_ *_x_* is slightly smaller than that in MoO_3‐_ *_x_* (as marked by the gray shaded area in Figure 4b). The lower Mo–Mo peak amplitude suggests that Ru addition perturbs the local Mo─Mo coordination environment, potentially through increased structural disorder [18, 46, 47, 48, 49]. To further elucidate the coordination environment, two‐dimensional (2D) and three‐dimensional (3D) wavelet‐transformed extended XAFS (WT‐EXAFS) analyses were performed for MoO_3‐_ *_x_* (Figure 4c,e), Ru‐MoO_3‐_ *_x_* (Figure 4d,f) and the reference Mo sample (Figure S2). In all samples, a strong and localized signal corresponding to Mo─O coordination is observed, suggesting the first coordination shell remains intact. In contrast, pronounced differences are observed in the Mo─Mo coordination region. The Mo─Mo signal in MoO_3‐_ *_x_* is sharp and intense, whereas in Ru‐MoO_3‐_ *_x_*‐3 it becomes noticeably weaker and broader (as indicated by the black circles in Figure 4e,f). These changes suggest that Ru addition perturbs the local Mo─Mo environment, primarily reflecting increased local structural disorder. The observed local structural perturbation may influence the electronic structure and is consistent with the experimentally measured increase in electrical conductivity. Concurrently, the increased local structural disorder may provide additional active sites, which is favorable for enhancing the electrical and electrochemical performance of the material.

**FIGURE 4:**
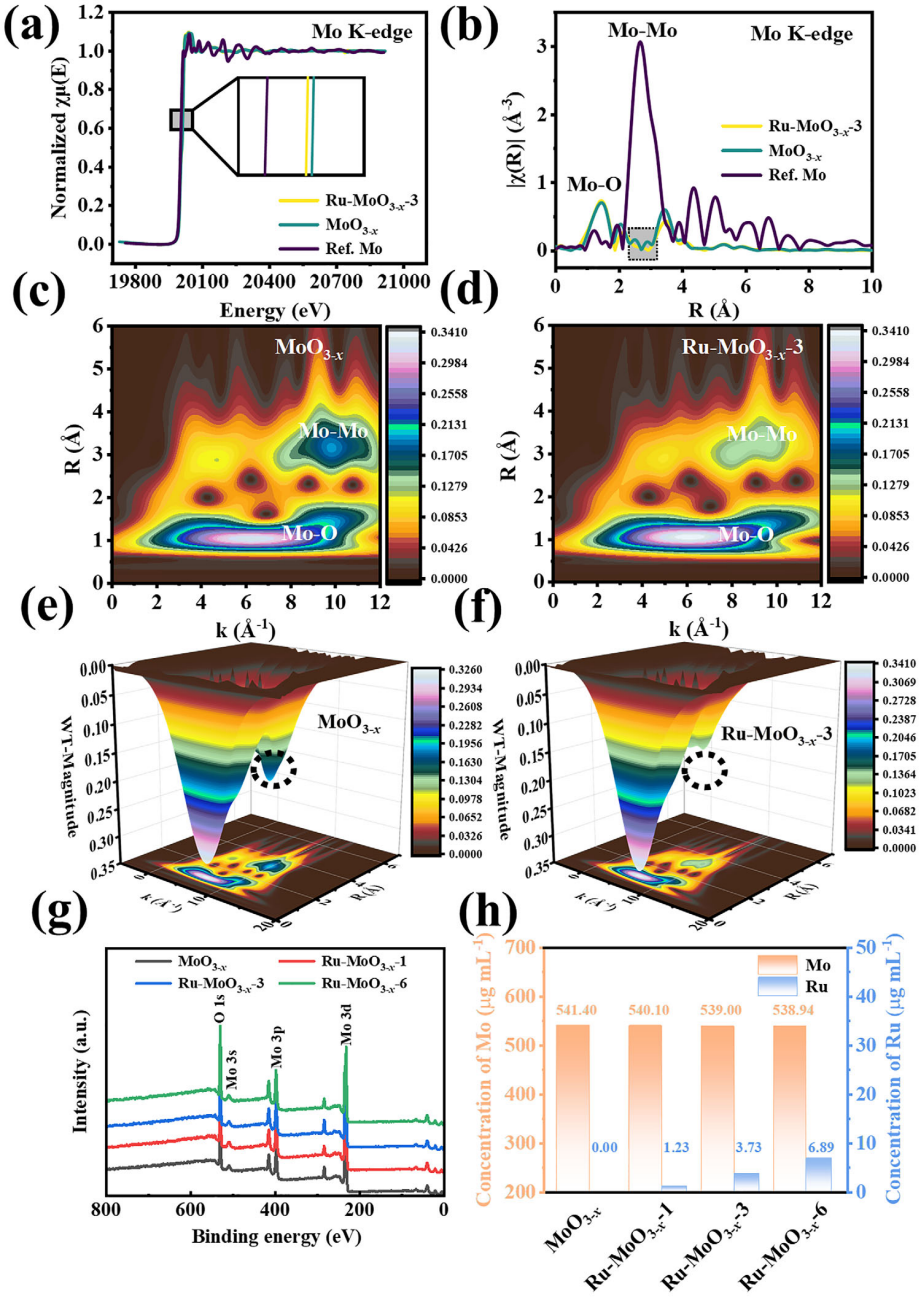
The Mo K‐edge (a) XANES and (b) FT‐EXAFS spectra of Ru‐MoO_3‐_ *_x_*‐3, MoO_3‐_ *_x_* and the reference Mo (Ref. Mo). WT‐EXAFS spectra of (c) MoO_3‐_ *_x_* and (d) Ru‐MoO_3‐_ *_x_*‐3, and the corresponding 3D images of (e) MoO_3‐_ *_x_* and (f) Ru‐MoO_3‐_ *_x_*‐3. (g) XPS survey spectra and (h) the ICP‐MS concentrations of MoO_3‐_ *_x_*, Ru‐MoO_3‐_ *_x_*‐1, Ru‐MoO_3‐_ *_x_*‐3 and Ru‐MoO_3‐_ *_x_*‐6.

Furthermore, the chemical compositions of all samples were analyzed using X‐ray photoelectron spectroscopy (XPS) and inductively coupled plasma mass spectrometry (ICP‐MS). The XPS survey spectrum (Figure 4g) confirms Mo and O; Ru may be below the survey detection limit. ICP‐MS results provide the quantitative concentrations of Mo and Ru. Among the Ru‐doped samples, the Ru content in Ru‐MoO_3‐_ *_x_*‐3 was determined by ICP‐MS to be 0.69 wt.% (Figure 4h). Such a low Ru loading is advantageous for reducing material cost. The introduction of Ru leads to a reduced average valence state of Mo and an increased concentration of oxygen vacancies in Ru‐MoO_3‐_ *_x_* as further corroborated by the high‐resolution Mo 3d and O 1s XPS spectra.

As shown in Figure 5a, the high‐resolution Mo 3d spectra of all samples were deconvoluted into two sets of spin‐orbit doublets. The dominant peaks located at approximately 232.7 and 235.9 eV are attributed to Mo^6+^ (Mo 3d_5/2_ and Mo 3d_3/2_), while the secondary peaks at around 231.0 and 235.0 eV correspond to Mo^5+^ species [20, 50]. Quantitative analysis reveals that the proportions of Mo^5+^ in the MoO_3‐_ *_x_*, Ru‐MoO_3‐_ *_x_*‐1, Ru‐MoO_3‐_ *_x_*‐3, and Ru‐MoO_3‐_ *_x_*‐6 are 3.2%, 3.3%, 8.6%, and 7.2%, respectively. Among these samples, Ru‐MoO_3‐_ *_x_*‐3 exhibits the highest Mo^5+^ fraction, further confirming that Ru introduction induces a more reduced Mo valence state, in good agreement with the XANES results. Similarly, the O 1s spectra (Figure 5b) reveal two components: lattice oxygen (∼530.6 eV) and oxygen vacancy‐related species (∼532.1 eV) [51]. The relative peak‐area fraction of oxygen‐vacancy‐related species (Tables S2–S5) is 6.2% for the pristine MoO_3‐_ *_x_*, and increases to 8.4%, 15.6%, and 10.7% for Ru‐MoO_3‐_ *_x_*‐1, Ru‐MoO_3‐_ *_x_*‐3, and Ru‐MoO_3‐_ *_x_*‐6, respectively. Consistent with the EPR results discussed earlier, in which the g = 2.0 signal intensity progressively increases in the order of MoO_3‐_ *_x_* < Ru‐MoO_3‐_ *_x_*‐1< Ru‐MoO_3‐_ *_x_*‐6< Ru‐MoO_3‐_ *_x_*‐3, the quantitative XPS results exhibit the same trend. Specifically, the relative peak‐area fraction of oxygen‐vacancy‐related species derived from the O 1s XPS spectra increases from 6.2% for MoO_3‐_ *_x_*, to 8.4% for Ru‐MoO_3‐_ *_x_*‐1, and 10.7% for Ru‐MoO_3‐_ *_x_*‐6, reaching a maximum of 15.6% for Ru‐MoO_3‐_ *_x_*‐3. To quantitatively evaluate this consistency, a scatter plot correlating the EPR signal intensity with the XPS‐derived O_v_‐related peak‐area fraction was constructed (Figure S3). The linear fit yields a high Pearson correlation coefficient of r = 0.88, indicating a positive correlation between the two independently measured defect‐related signals. The agreement between surface‐sensitive XPS and unpaired‐electron‐sensitive EPR support the conclusion that the optimal Ru doping level (Ru‐MoO_3‐_ *_x_*‐3) results in the highest oxygen‐vacancy concentration among the nanostructured films examined. Collectively, all Ru‐doped samples exhibit higher oxygen‐vacancy concentrations than pristine MoO_3‐_ *_x_*, demonstrating that Ru introduction effectively modulates the defect structure of MoO_3‐_ *_x_*.

**FIGURE 5:**
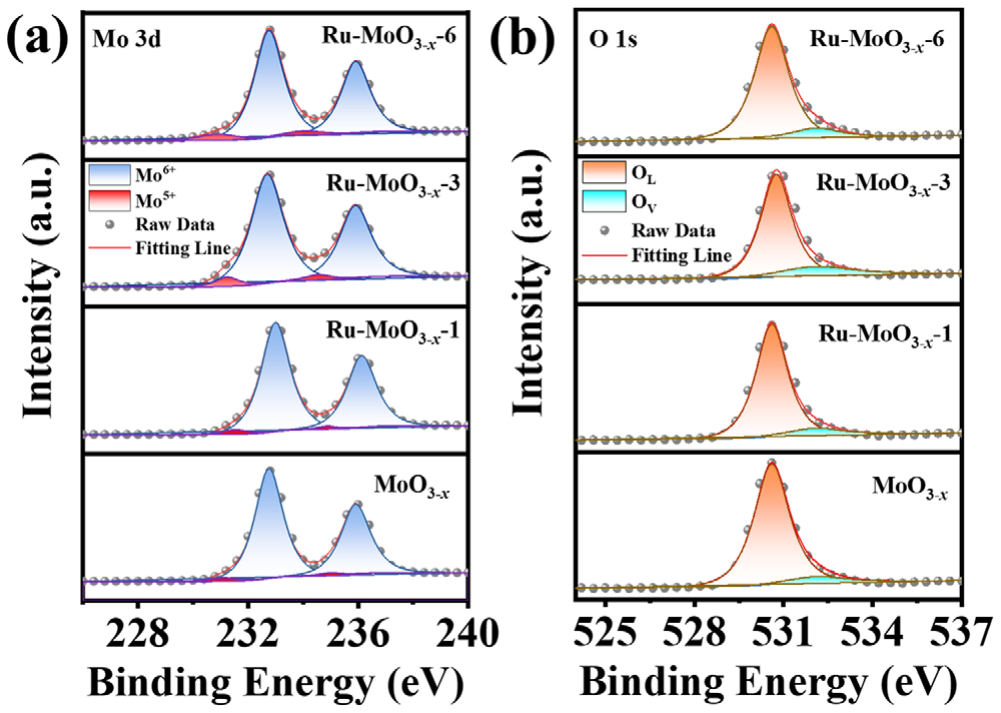
High‐resolution XPS spectra of the MoO_3‐_ *_x_*, Ru‐MoO_3‐_ *_x_*‐1, Ru‐MoO_3‐_ *_x_*‐3, and Ru‐MoO_3‐_ *_x_*‐6 films: (a) Mo 3d, and (b) O 1s.

Ultraviolet‐visible (UV‐vis) spectroscopy and ultraviolet photoelectron spectroscopy (UPS) were used to further analyze the electronic structure and optical bandgap of the MoO_3‐_ *_x_* and Ru‐MoO_3‐_ *_x_* flexible films with various Ru contents. As shown in the UV‐vis absorption spectra (Figure 6a), all flexible films exhibit strong absorption in the ultraviolet region (200–400 nm). The pronounced absorption tail near the band edge indicates the presence of defect‐induced electronic states within the materials. Based on the UV‐vis absorption data, Tauc plots (Figure S4) were constructed using the Tauc equation, *(αhν)^n^ = A(hν—E_g_)* [52], and the optical bandgaps for the flexible films were determined by linear extrapolation. Pristine MoO_3‐_ *_x_* exhibits an optical bandgap of 2.94 eV (E_g1_), which progressively narrows to 2.87 eV (E_g2_) for Ru‐MoO_3‐_ *_x_*‐1, 2.70 eV (E_g3_) for Ru‐MoO_3‐_ *_x_*‐3, and 2.80 eV (E_g4_) for Ru‐MoO_3‐_ *_x_*‐6. This trend suggests that moderate Ru incorporation introduces impurity or defect‐related states within the band structure, thereby reducing the optical transition energy. Ultraviolet photoelectron spectroscopy (UPS) measurements revealed the secondary electron cutoff edge (E_cutoff_) and valence band maximum position (E_v_) for the films. Furthermore, the work function (W_f_) and ionization energy (IE) of MoO_3‐_ *_x_*, Ru‐MoO_3‐_ *_x_*‐1, Ru‐MoO_3‐_ *_x_*‐3 and Ru‐MoO_3‐_ *_x_*‐6 were determined from the UPS spectra (Figures 6b‐c). The work function was calculated using the equation W_f_ = *hν*‐(E_cutoff_—E_f_), where *hν* = 21.22 eV is the photon energy of the He I excitation source, E_cutoff_ is the secondary electron cutoff energy, and E_f_ is the Fermi level. Subsequently, the ionization energy was obtained using the relation IE = W_f_+(E_f_‐E_v_), where E_v_ represents the valence band maximum. As summarized in Figure 6d, the corresponding W_f_ values (with respect to vacuum) are 3.87, 3.73, 3.45, and 3.68 eV, while the IE values are 6.72, 6.47, 6.14, and 6.39 eV, for MoO_3‐_ *_x_*, Ru‐MoO_3‐_ *_x_*‐1, Ru‐MoO_3‐_ *_x_*‐3, and Ru‐MoO_3‐_ *_x_*‐6, respectively. The work function reflects the energy required for an electron to escape from the Fermi level to the vacuum, while the ionization energy reflects the binding energy of valence electrons relative to the vacuum level. Ru doping leads to a simultaneous reduction in both the work function and ionization energy of MoO_3‐_ *_x_*. Notably, the Ru‐MoO_3‐_ *_x_*‐3 film exhibits the lowest values for both parameters, with the work function (W_f_) decreasing from 3.87 eV (pristine MoO_3‐_ *_x_*) to 3.45 eV, and its ionization energy (IE) dropping from 6.72 to 6.14 eV. The concurrent reduction in both the work function and ionization energy demonstrates that Ru doping not only modulates the electronic states near the Fermi level but also alters the valence band structure, thereby achieving effective control over the energy‐level positions. As illustrated in the energy‐band schematic diagram (Figure 6e), both the Fermi level and valence band maximum of the Ru‐doped samples shift upward significantly, providing a direct physical origin for the enhanced electrical conductivity.

**FIGURE 6:**
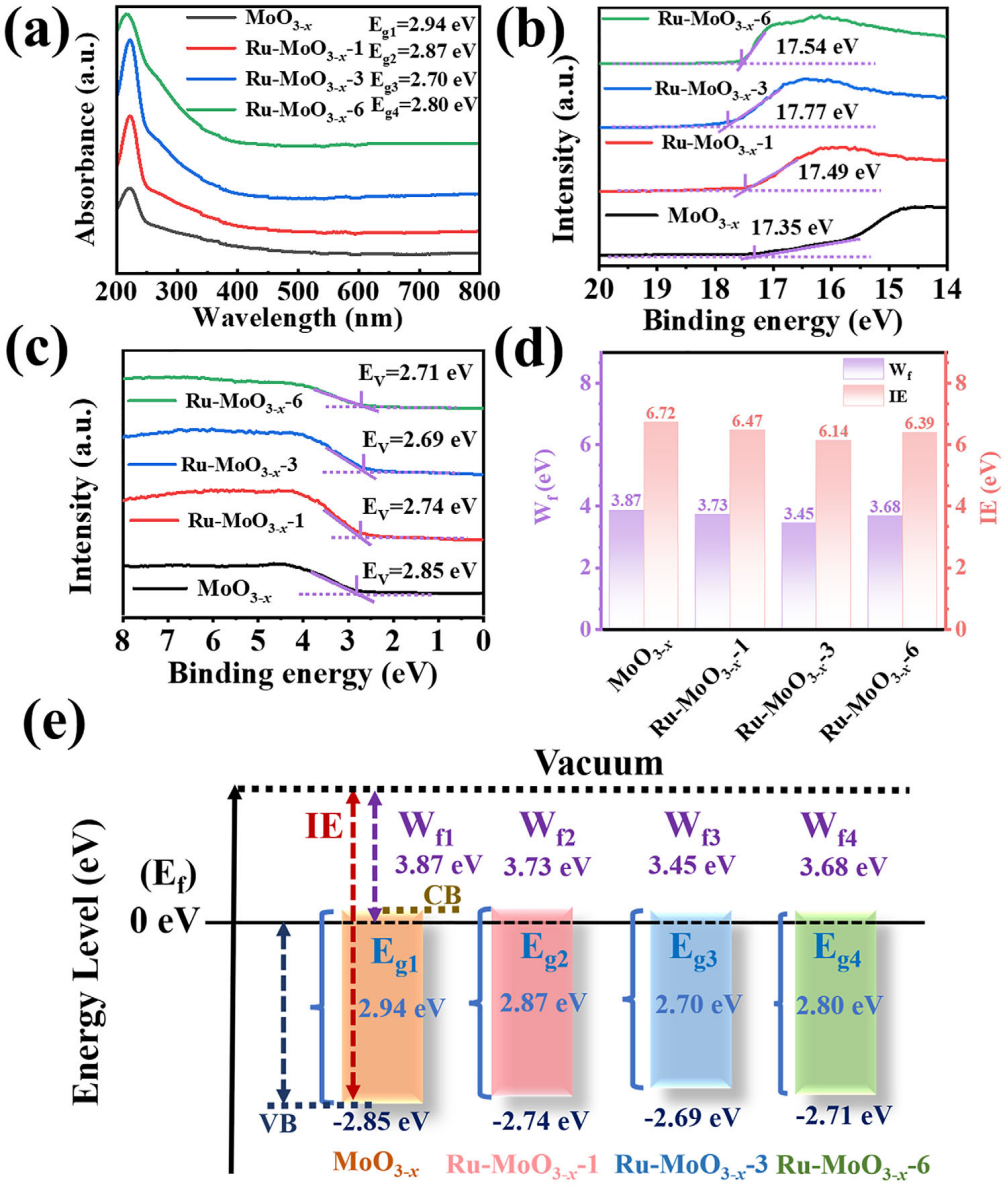
(a) Ultraviolet‐visible (UV‐vis) absorption spectra, (b) secondary‐electron cutoff edge (E_cutoff_) and (c) valence band maximum position (E_v_) in the UPS spectra, (d) W_f_ and IE, and (e) energy band schematic diagram of MoO_3‐_ *_x_*, and Ru‐MoO_3‐_ *_x_* flexible films with varying Ru concentrations.

To further understand the electronic structure and orbital interactions of MoO_3‐_ *_x_* and Ru‐MoO_3‐_ *_x_* films, density functional theory (DFT) calculations were performed. Figure S5a illustrates the atomic structural models of MoO_3‐_ *_x_* and Ru‐MoO_3‐_ *_x_*. The calculated electronic band structures along the high‐symmetry paths in the Brillouin zone are Γ‐X‐S‐Y‐Γ‐Z‐U‐R for MoO_3‐_ *_x_* and Γ‐X‐S‐Y‐Γ‐Z‐U‐R‐T for Ru‐MoO_3‐_ *_x_* (Figure 7a,b). For Ru‐MoO_3‐_ *_x_*, the valence‐band maximum is located at the S point, whereas the conduction‐band minimum resides at the Γ point [17, 37]. The band structure analysis reveals that the introduction of Ru induces a downward shift of the conduction band minimum. This effectively reduces the band gap and corresponds to an expansion of occupied states near the Fermi level, theoretically suggesting an enhanced intrinsic electrical conductivity. Figure 7c shows the total density of states (TDOS) for MoO_3‐_ *_x_* and Ru‐MoO_3‐_ *_x_* films. Further insights are gained from the partial density of states (PDOS) analysis. In Ru‐MoO_3‐_ *_x_*, the Mo d‐PDOS intensity near the Fermi level is markedly reduced (Figure 7d). This decrease indicates a possible weakening of direct Mo─Mo interactions, consistent with the XAFS results discussed above, which suggest increased disorder in the local Mo─Mo environment upon Ru addition. The orbital‐resolved PDOS further shows contributions from Mo d, O p, and Ru d states (Figure 7d–f), with a substantial contribution from Ru d states near the Fermi level. Together with the calculated band structures, these results indicate Ru‐associated electronic‐structure modification consistent with the reduced bandgap. Moreover, UV‐vis and UPS analyses reveal a narrowed optical bandgap and a substantially reduced work function, respectively. Collectively, these experimental results and DFT calculations provide a possible electronic explanation for the measured conductivity enhancement in the Ru‐MoO_3‐_ *_x_* films.

**FIGURE 7:**
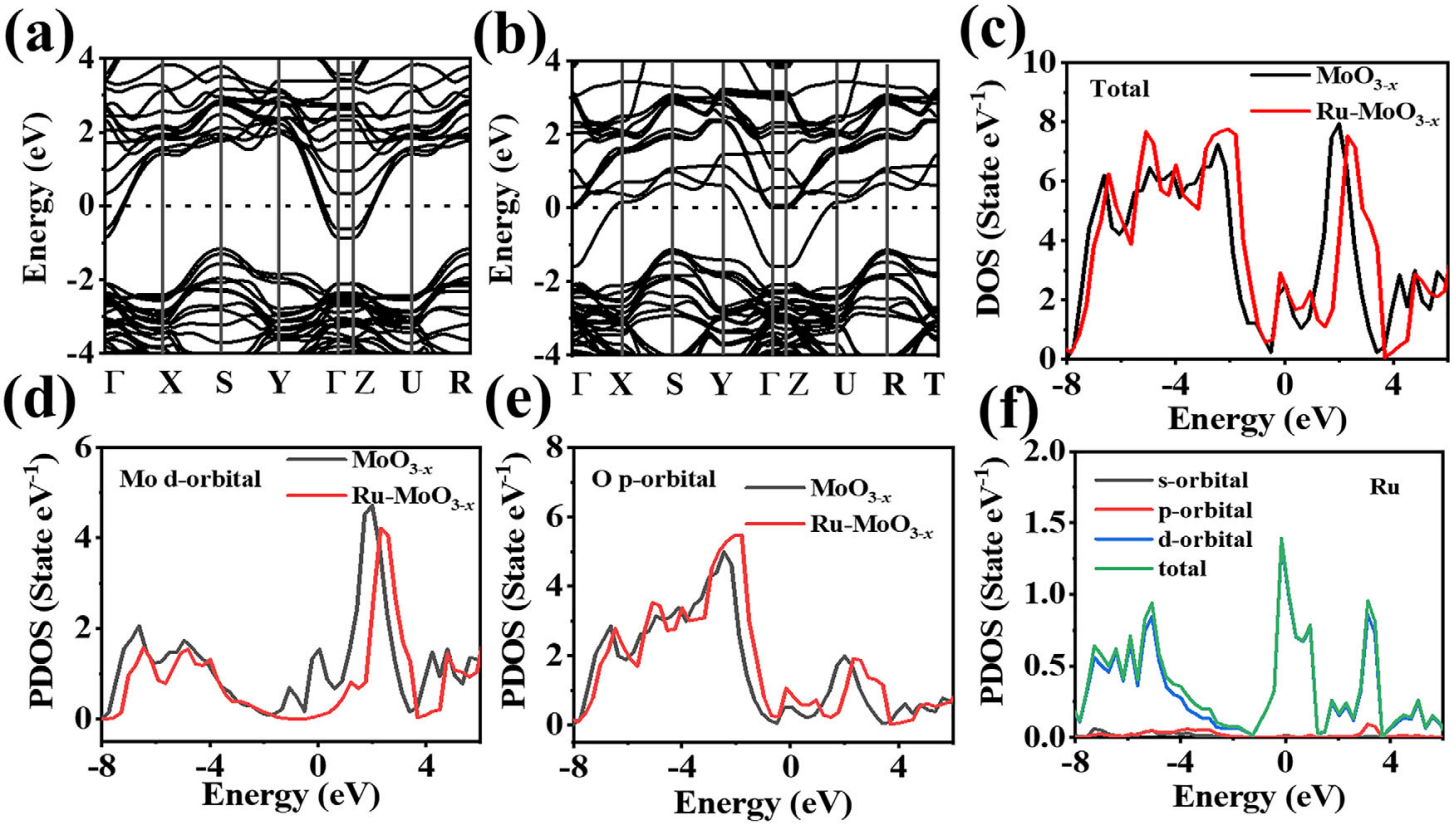
Electronic structure of MoO_3‐_ *_x_* and Ru‐MoO_3‐_ *_x_*. (a, b) Band structures. (c) Total DOS. (d, e) PDOS of Mo d‐ and O p‐orbitals. (f) PDOS of Ru d‐orbitals in Ru‐MoO_3‐_ *_x_*.

The electrochemical performance of flexible MoO_3‐_ *_x_* and Ru‐doped MoO_3‐_ *_x_* (Ru‐MoO_3‐_ *_x_*) film electrodes was investigated in a three‐electrode configuration using on a CHI 660E electrochemical workstation. All tests were conducted in 3 M H_2_SO_4_ electrolyte with an Hg/Hg_2_SO_4_ reference electrode within a potential window of −0.95 to −0.15 V. As depicted in the cyclic voltammetry (CV) and galvanostatic charge‐discharge (GCD) curves (Figure 8a,b), the pronounced redox peaks and charge‐discharge plateaus originate from the Mo^5+^/Mo^6+^ redox couple associated with reversible H^+^ intercalation. Notably, the Ru‐MoO_3‐_ *_x_* electrodes exhibit a substantially larger CV‐integrated area and longer discharge times than pristine MoO_3‐_ *_x_*, indicating a significantly enhanced specific capacitance. The performance enhancement is further corroborated by electrochemical impedance spectroscopy (EIS) results (Figure 8c), which reveal a smaller internal resistance for the Ru‐doped films. Figure 8d,e displays the specific capacitance values of the MoO_3‐_ *_x_*, Ru‐MoO_3‐_ *_x_*‐1, Ru‐MoO_3‐_ *_x_*‐3, and Ru‐MoO_3‐_ *_x_*‐6 film electrodes measured at various scan rates and current densities. At a scan rate of 2 mV s^−1^, the specific capacitances are 815.6 F g^−1^ for MoO_3‐_ *_x_*, 1331.2 F g^−1^ for Ru‐MoO_3‐_ *_x_*‐1, 2034.6 F g^−1^ for Ru‐MoO_3‐_ *_x_*‐3, and 1646.7 F g^−1^ for Ru‐MoO_3‐_ *_x_*‐6. At a current density of 1 A g^−1^, the values are 916.5, 1382.5, 2047.5, and 1666.2 F g^−1^, respectively. Notably, the Ru‐MoO_3‐_ *_x_*‐3 electrode delivers the highest capacitance, reaching 2034.6 F g^−1^ at 2 mV s^−1^ and 2047.5 F g^−1^ at 1 A g^−1^. These values substantially surpass those of most previously reported MoO_3_‐based materials (Table 1) [15, 16, 19, 23, 24, 53, 54] and approach the theoretical specific capacitance of MoO_3_ (2700 F g^−1^), which can be attributed to the high oxygen‐vacancy concentration and enhanced electrical conductivity. The CV and GCD curves of the Ru‐MoO_3‐_ *_x_*‐3 electrode are shown in Figures 8f,g. Moreover, the optimized Ru‐MoO_3‐_ *_x_*‐3 electrode demonstrates excellent long‐term cycling stability, retaining 95.2% of its initial capacitance after 5000 cycles at 2 A g^−1^ (Figure S6). To elucidate the origin of the high performance, the charge‐storage mechanism of the electrode material was investigated. Based on the electrochemical kinetic analysis (Equations S3 and S4), fitting both the cathodic and anodic peak currents at scan rates of 2, 5, 10, and 20 mV s^−1^ yields b‐values of approximately 0.5 for both processes (Figure 8h). This indicates that the charge storage is predominantly governed by a diffusion‐controlled process, characteristic of battery‐type behavior. To quantify these contributions, the capacitive and diffusion currents were deconvoluted using the Dunn equations (Equations S5 and S6). As shown in Figure 8i, diffusion‐controlled processes dominate at low scan rates, contributing 90.0% at 2 mV s^−1^ and 75.9% at 20 mV s^−1^. The diffusion dominance is in excellent agreement with the b‐value analysis and directly accounts for the high capacitance, which is attributed to substantial H^+^ intercalation coupled with the Mo^5+^/Mo^6+^ redox reaction. In contrast, at a high scan rate of 100 mV s^−1^, the diffusion contribution decreases to 18.2%, and the surface‐capacitive process becomes dominant, enabling rapid charge transfer. The synergistic combination of high‐capacitance diffusion‐controlled storage and fast surface processes underpins the robust electrochemical performance of the material.

**FIGURE 8:**
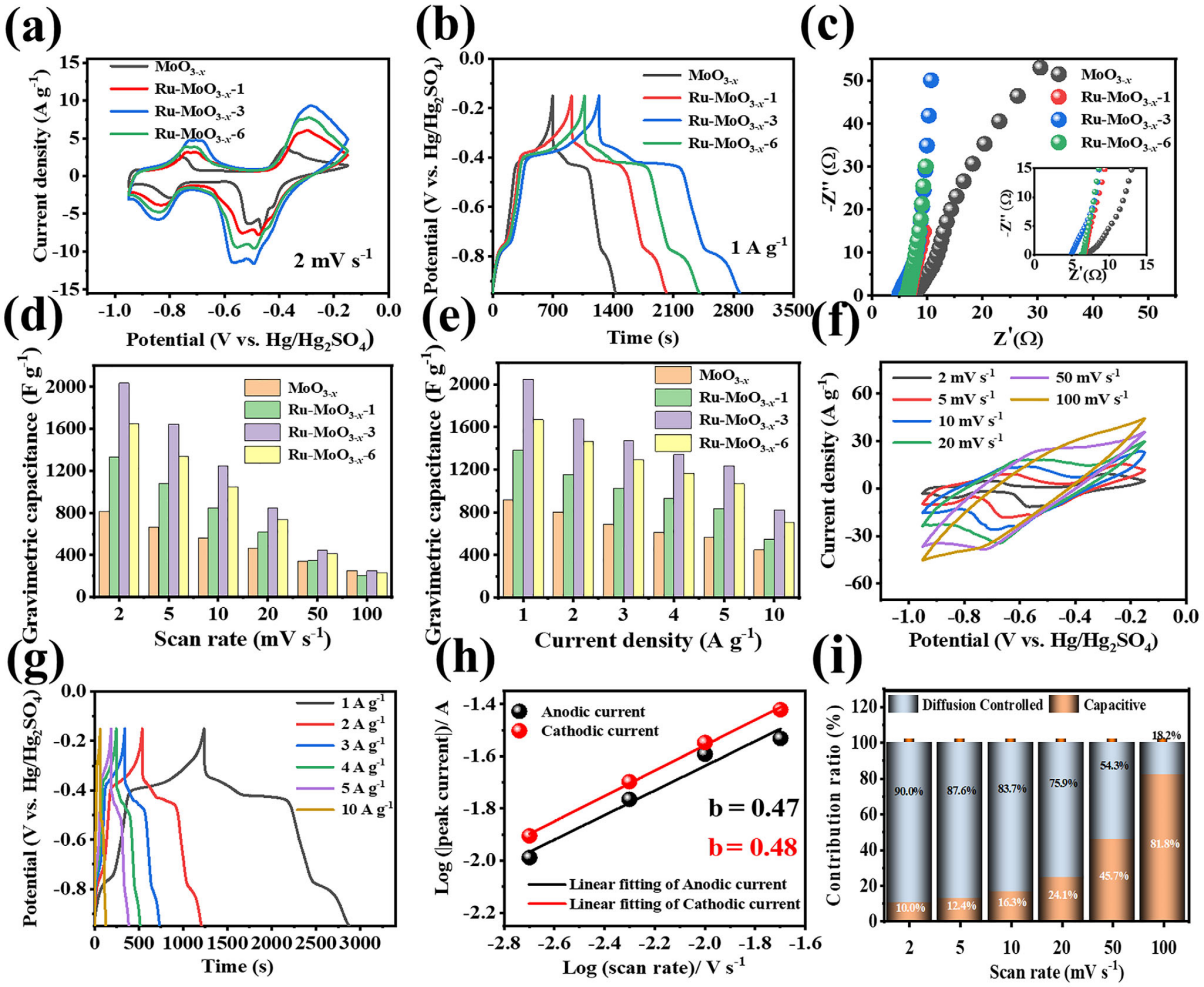
Electrochemical performance for MoO_3‐_ *_x_* and Ru‐MoO_3‐_ *_x_* electrodes with varying Ru concentrations: (a) CV curves, (b) GCD curves, (c) Nyquist impedance plots (the inset corresponds to the magnified high‐frequency region), (d) gravimetric capacitance at different scan rates, (e) gravimetric capacitance at various current densities. Electrochemical performance for Ru‐MoO_3‐_ *_x_*‐3 electrode: (f) CV curves, (g) GCD curves, (h) log *i_peak_* versus log *v* from 2 to 20 mV s^−1^. (i) Capacitive‐ and diffusion‐controlled contributions to the CV current at different scan rates.

**TABLE 1:** Comparison of the electrochemical performance for MoO_3_‐based electrodes.

Electrodes	Electrolyte	Potential window	Specific capacitance	Cycling stability	Flexible film	Refs.
MoO_3‐_ *_x_* nanosheets	1 M LiClO_4_	−0.55 ∼ 0.5 vs. (Ag/AgCl)/V	80.0 F g^−1^ at 1 A g^−1^	1500 (76.8%)	No	[15]
MoO_3_ nanosheets	1 M KOH	−0.6 ∼ 0.2 vs. (Hg/Hg_2_Cl_2_)/V	829 F g^−1^ at 2 A g^−1^	2000 (91.0%)	No	[16]
MoO_3_ nanoplates	1 M H_2_SO_4_	−0.3 ∼ 0.4 vs. (Ag/AgCl)/V	994.2 F g^−1^ at 0.5 A g^−1^	1500 (84.0%)	No	[19]
OV‐MoO_3_/Ce ultrathin nanoflakes	1 M H_2_SO_4_	−0.6 ∼ 0.2 vs. (Hg/Hg_2_Cl_2_)/V	1446.3 F g^−1^ at 5 A g^−1^	5000 (94.8%)	No	[23]
S‐MoO_3‐_ *_x_*	5 M LiCl	−1.0 ∼ 0 vs (Hg/Hg_2_Cl_2_)/ V	249.8 F g^−1^ at 0.5 mA cm^−2^	5000 (90.0%)	No	[24]
MoO_3_ film	2 M Li_2_SO_4_	−1.0 ∼ 0.0 vs (Hg/Hg_2_Cl_2_)/V	835.0 F g^−1^ at 1 A g^−1^	8000 (98%)	Yes	[53]
MoO*_x_* nanobelts	HClO_4_/NaClO_4_	−1 ∼‐0.2 vs (Ag/AgCl)/V	448.0 F g^−1^ at 1 A g^−1^	20000 (86.0%)	No	[54]
MoO_3‐_ *_x_*	3 M H_2_SO_4_	−0.95 ∼ −0.15 vs. (Hg/Hg_2_SO_4_)/V	916.5 F g^−1^ at 1 A g^−1^	—	Yes	This work
Ru‐MoO_3‐_ *_x_*‐1	3 M H_2_SO_4_	−0.95 ∼ −0.15 vs. (Hg/Hg_2_SO_4_)/V	1382.5 F g^−1^ at 1 A g^−1^	—	Yes	
Ru‐MoO_3‐_ *_x_*‐3	3 M H_2_SO_4_	−0.95 ∼ −0.15 vs. (Hg/Hg_2_SO_4_)/V	2047.5 F g^−1^ at 1 A g^−1^	5000 (95.2%)	Yes	
Ru‐MoO_3‐_ *_x_*‐6	3 M H_2_SO_4_	−0.95 ∼ −0.15 vs. (Hg/Hg_2_SO_4_)/V	1666.2 F g^−1^ at 1 A g^−1^	—	Yes	

The optimal Ru‐MoO_3_ *_‐x_*‐3 flexible film exhibits an exceptional specific capacitance of 2047.5 F g^−1^, which approaches the theoretical capacitance of molybdenum trioxide. To demonstrate the practical versatility of this material in energy storage platforms, asymmetric supercapacitors (ASCs) were assembled utilizing Ru‐MoO_3_ *_‐x_*‐3 as the negative electrode and activated carbon (AC) as the positive electrode. The electrochemical performance of these devices (denoted as Ru‐MoO_3_ *_‐x_*‐3//AC) was systematically evaluated in three distinct electrolyte systems: aqueous 3 M H_2_SO_4_, quasi‐solid‐state PVA‐H_2_SO_4_ hydrogel, and an organic ionic liquid electrolyte consisting of 1 M EmimTFSI/LiTFSI in acetonitrile (ACN). As illustrated in Figure 9a,b, the CV curves (at 2 mV s^−1^) and GCD profiles (at 1 A g^−1^) reveal the distinct electrochemical behaviors of the Ru‐MoO_3_ *_‐x_*‐3//AC devices in these diverse ionic environments. In acidic media (both aqueous and hydrogel electrolytes), the devices operate within a voltage window of 0–1.6 V, and exhibit prominent redox peaks. These features are ascribed to the effective utilization of abundant active sites for reversible Mo^5+^/Mo^6+^ redox transitions coupled with rapid proton (H^+^) intercalation kinetics. Conversely, the organic electrolyte system enables a significantly widened operating voltage window of 2.4 V, demonstrating exceptional high‐voltage stability. However, the CV profiles in the organic medium appear quasi‐rectangular with negligible redox peaks. This discrepancy in electrochemical behavior implies fundamentally different charge storage mechanisms dictated by ionic radii and solvation effects [55, 56, 57]. In the aqueous 3 M H_2_SO_4_ system, the extremely small radius of protons (H^+^) and their rapid Grotthuss hopping mechanism through free water enable facile proton intercalation into the Ru‐MoO_3‐_ *_x_* lattice, yielding maximum pseudocapacitance [58, 59]. In the quasi‐solid‐state PVA‐H_2_SO_4_ hydrogel, the fundamental proton intercalation mechanism is retained; however, the solvation environment transitions from free water to a polymer‐bound hydrogen network. This increases the steric hindrance and diffusion resistance, slightly slowing down the kinetics but securing the structural integrity of the flexible device. In stark contrast, the charge storage mechanism in the organic ionic liquid (1 M EmimTFSI/LiTFSI in ACN) is fundamentally limited by ionic size and desolvation penalties. The large effective ionic sizes of the bulky Emim^+^ and TFSI^−^ ions likely limit their access to narrow interlayer sites, contributing mainly to electrical double‐layer capacitance (EDLC) [60]. Although Li^+^ possesses a smaller bare radius, it is heavily solvated by ACN molecules. Overcoming the substantial desolvation energy barrier to strip the ACN solvation sheath significantly restricts the Li^+^ insertion kinetics into the host lattice [61]. Consequently, while the distinct redox peaks are suppressed and capacitance is reduced due to these structural and solvation constraints, the non‐aqueous environment effectively expands the stable voltage window. The Ragone plot in Figure 9c compares the energy and power densities of these devices. The ASCs deliver maximum energy densities of 40.9 Wh kg^−1^ (in 3 M H_2_SO_4_), 39.8 Wh kg^−1^ (in PVA‐H_2_SO_4_), and 25.3 Wh kg^−1^ (in organic electrolyte). Additionally, the maximum power densities are 4000 W kg^−1^ for the acidic systems (H_2_SO_4_ and PVA‐H_2_SO_4_) and 6000 W kg^−1^ for the organic system (EmimTFSI/LiTFSI). These results indicate that the Ru‐MoO_3_ *_‐x_*‐3 electrode is compatible with different electrolyte systems, allowing for either high‐voltage operation (in organic electrolytes) or high energy density (in acidic media). This adaptability enables the fabrication of devices optimized for specific performance requirements. Notably, the quasi‐solid‐state device retains competitive performance despite the inherently restricted ionic mobility within the polymer gel matrix. Beyond electrochemical metrics, the mechanical robustness of the quasi‐solid‐state device was rigorously evaluated. As shown in Figure 9d,e, the CV curves remain virtually identical over bending angles ranging from 0° to 180°. The strong overlap of CV curves attests to the excellent flexibility and fatigue resistance of the Ru‐MoO_3_ *_‐x_*‐3 electrode, confirming that its structural integrity is preserved even under severe deformation. To demonstrate proof‐of‐concept applicability, the Ru‐MoO_3_ *_‐x_*‐3//AC devices were integrated into a circuit (Figure 9f), successfully illuminating red, blue, and green light‐emitting diodes (LEDs) for over 3 min and stably powering an alarm clock for over 10 min (Supporting Information: S1‐S4, Videos). These demonstrations confirm that the Ru‐MoO_3_ *_‐x_*‐3 flexible film is a promising candidate for both high‐voltage energy storage and flexible wearable electronics.

**FIGURE 9:**
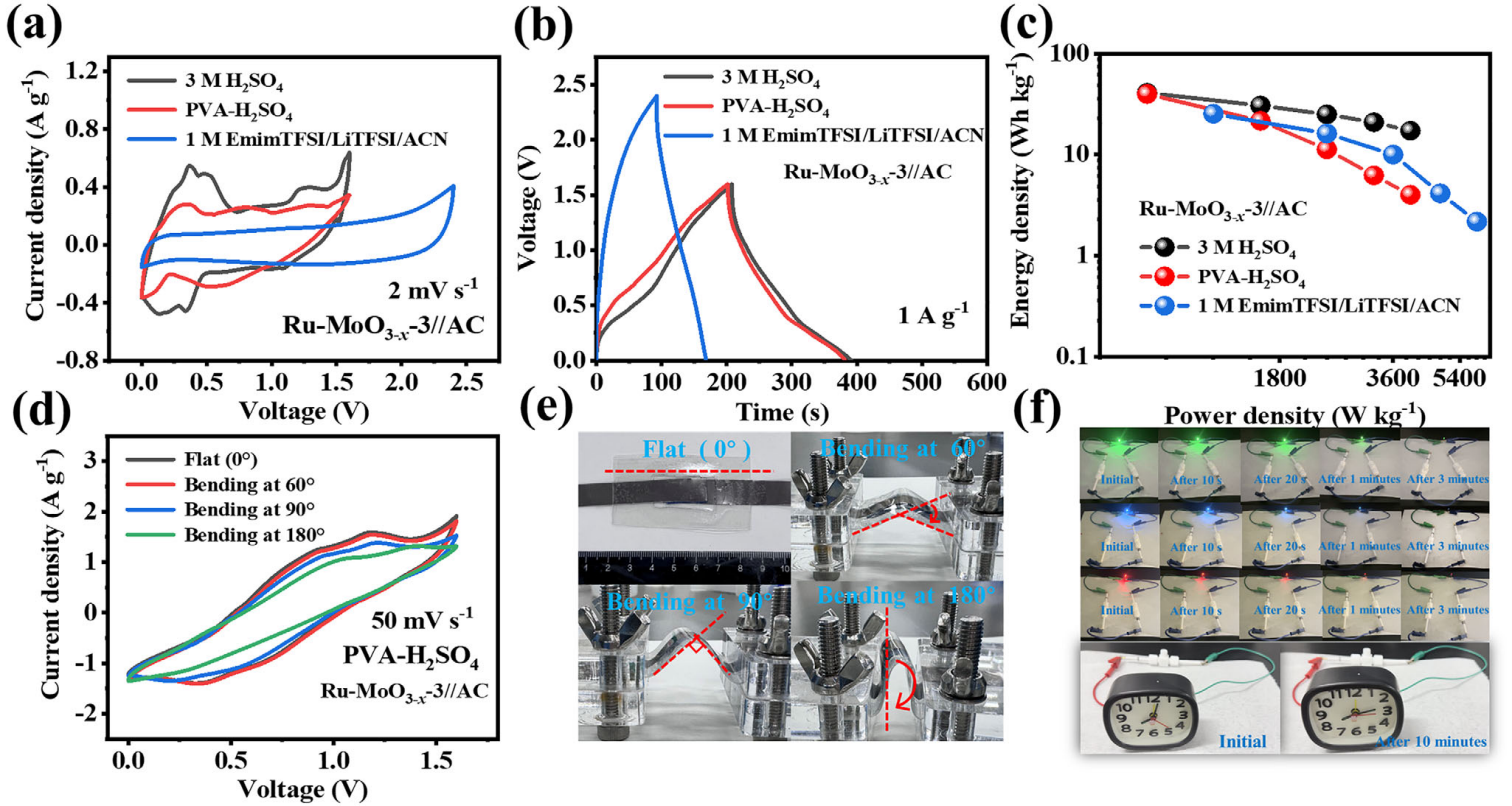
Electrochemical performance of the Ru‐MoO_3‐_ *_x_*‐3//AC device in different electrolytes: (a) CV curves, (b) GCD curves, (c) Ragone plots, (d) CV curves of the Ru‐MoO_3‐_ *_x_*‐3//AC quasi‐solid‐state device at different bending angles at a scan rate of 50 mV s^−1^. (e) Photographs of the Ru‐MoO_3‐_ *_x_*‐3//AC quasi‐solid‐state device at different bending angles (0°, 60°, 90°, 180°). (f) Photographs of the Ru‐MoO_3‐_ *_x_*‐3//AC device in a two‐electrode cell lighting up red, blue, and green LEDs and powering an alarm clock in 3 M H_2_SO_4_ electrolyte.

## 3 Conclusions

In summary, a high‐performance flexible Ru‐doped MoO_3_ *_‐x_* film electrode was successfully fabricated via a facile one‐step solvothermal strategy. Experimental characterization confirms the presence of Ru in the films and indicates that Ru addition is accompanied by local structural disorder and oxygen‐vacancy enrichment. UV‐vis/UPS measurements and DFT calculations further indicate a narrowed optical bandgap, a reduced work function, and modified electronic states near the Fermi level, changes that are consistent with the enhanced electrical conductivity and electrochemical performance of Ru‐MoO_3‐_ *_x_*.

Benefiting from the synergistic effects of the mixed nanowire–nanobelt morphology and optimized electronic properties, the optimal Ru‐MoO_3_ *_‐x_*‐3 electrode exhibits a near‐theoretical specific capacitance of 2047.5 F g^−1^. Its charge‐storage mechanism is dominated by diffusion‐controlled processes. The assembled asymmetric supercapacitors demonstrate remarkable adaptability, delivering superior energy density (40.9 Wh kg^−1^) in acidic electrolytes and exceptional high‐voltage stability (2.4 V) in organic systems. Importantly, this electrolyte‐dependent behavior provides a general design principle for future device engineering: acidic electrolytes preferentially promote high specific capacitance and energy density, whereas suitable organic electrolytes are highly effective for extending the operating voltage window, enabling targeted optimization of flexible energy‐storage devices for different application scenarios. Moreover, the quasi‐solid‐state device maintains stable electrochemical performance under bending angles from 0° to 180°, proving its potential for practical wearable applications. This work not only presents a superior electrode material for flexible supercapacitors but also offers a viable strategy for tuning the electronic structure of transition metal oxides via doping‐mediated defect engineering.

## 4 Experimental Section

The experimental details, including electrode preparation, material characterization, electrochemical measurements, and calculations, are available in the Supporting Information.

## Conflicts of Interest

The authors declare no conflict of interest.

## Supporting information




**Supporting File 1**: advs77131‐sup‐0001‐SuppMat.docx.

**Supporting File 2**: advs77131‐sup‐0002‐VideoS1.mp4.

**Supporting File 3**: advs77131‐sup‐0003‐VideoS2.mp4.

**Supporting File 4**: advs77131‐sup‐0004‐VideoS3.mp4.

**Supporting File 5**: advs77131‐sup‐0005‐VideoS4.mp4.

## Data Availability

The data that support the findings of this study are available from the corresponding author upon reasonable request.

## References

[1] N. Choudhary , C. Li , J. Moore , et al., “Asymmetric Supercapacitor Electrodes and Devices,” Advanced Materials 29, no. 21 (2017): 1605336, 10.1002/adma.201605336. 28244158

[2] M. Zhi , C. Xiang , J. Li , M. Li , and N. Wu , “Nanostructured carbon–metal oxide composite electrodes for supercapacitors: A review,” Nanoscale 5, no. 1 (2013): 72–88, 10.1039/C2NR32040A. 23151936

[3] Y. Huang , M. Zhu , Y. Huang , et al., “Multifunctional Energy Storage and Conversion Devices,” Advanced Materials 28, no. 38 (2016): 8344–8364, 10.1002/adma.201601928. 27434499

[4] Q. Zhu , D. Zhao , M. Cheng , et al., “A New View of Supercapacitors: Integrated Supercapacitors,” Advanced Energy Materials 9, no. 36 (2019): 1901081, 10.1002/aenm.201901081.

[5] D. Ge , L. Yang , L. Fan , et al., “Foldable Supercapacitors from Triple Networks of Macroporous Cellulose Fibers, Single‐Walled Carbon Nanotubes and Polyaniline Nanoribbons,” Nano Energy 11 (2015): 568–578, 10.1016/j.nanoen.2014.11.023.

[6] K. Qin , J. Kang , J. Li , et al., “Free‐Standing Porous Carbon Nanofiber/Ultrathin Graphite Hybrid for Flexible Solid‐State Supercapacitors,” ACS Nano 9, no. 1 (2015): 481–487, 10.1021/nn505658u. 25567451

[7] H. Cheng , C. Hu , Y. Zhao , and L. Qu , “Graphene Fiber: A New Material Platform for Unique Applications,” NPG Asia Materials 6, no. 7 (2014): 113, 10.1038/am.2014.48.

[8] C. Yang , Y. Tang , Y. Tian , et al., “Flexible Nitrogen‐Doped 2D Titanium Carbides (MXene) Films Constructed by an Ex Situ Solvothermal Method With Extraordinary Volumetric Capacitance,” Advanced Energy Materials 8, no. 31 (2018): 1802087, 10.1002/aenm.201802087.

[9] S. Zhang and N. Pan , “Supercapacitors Performance Evaluation,” Advanced Energy Materials 5, no. 6 (2015): 1401401, 10.1002/aenm.201401401.

[10] G. Wang , L. Zhang , and J. Zhang , “A Review of Electrode Materials for Electrochemical Supercapacitors,” Chemical Society Reviews 41, no. 2 (2012): 797–828, 10.1039/C1CS15060J. 21779609

[11] M. Winter and R. J. Brodd , “What Are Batteries, Fuel Cells, and Supercapacitors?,” Chemical Reviews 104, no. 10 (2004): 4245–4270, 10.1021/cr020730k. 15669155

[12] Y. Jia and Y. Ma , “Advances in MoO3‐Based Supercapacitors for Electrochemical Energy Storage,” Journal of Energy Storage 85 (2024): 111103, 10.1016/j.est.2024.111103.

[13] Q. Xue , J. Sun , Y. Huang , et al., “Recent Progress on Flexible and Wearable Supercapacitors,” Small 13, no. 45 (2017): 1701827, 10.1002/smll.201701827. 28941073

[14] Q. Jiang , N. Kurra , M. Alhabeb , Y. Gogotsi , and H. N. Alshareef , “All Pseudocapacitive MXene‐RuO_2_ Asymmetric Supercapacitors,” Advanced Energy Materials 8, no. 13 (2018): 1703043, 10.1002/aenm.201703043.

[15] B. Liu , S. Wu , Y. Lv , et al., “Facile Synthesis of Oxygen‐Deficient MoO_3‐x_ Nanosheets by Light Radiation for Fast Electrochromic Supercapacitors,” Electrochimica Acta 464 (2023): 142894, 10.1016/j.electacta.2023.142894.

[16] M. Kundu , D. Mondal , I. Mondal , et al., “A Rational Preparation Strategy of Phase Tuned MoO_3_ Nanostructures for High‐Performance All‐Solid Asymmetric Supercapacitor,” Journal of Energy Chemistry 87 (2023): 192–206, 10.1016/j.jechem.2023.08.014.

[17] X. Xiao , Z. Peng , C. Chen , et al., “Freestanding MoO_3_− Nanobelt/Carbon Nanotube Films for Li‐ion Intercalation Pseudocapacitors,” Nano Energy 9 (2014): 355–363, 10.1016/j.nanoen.2014.08.001.

[18] D. Feng , P. Wang , R. Qin , et al., “Flower‐Like Amorphous MoO_3−x_ Stabilized Ru Single Atoms for Efficient Overall Water/Seawater Splitting,” Advanced Science 10, no. 18 (2023): 2300342, 10.1002/advs.202300342. PMC10288252 37092569

[19] Y. Zhu , Y. Tan , and H. Li , “MoO_3_ Nanoplates Preparation via Self‐Sacrifice C_3_N_4_ for Supercapacitors in an Acid Electrolyte,” Journal of Energy Storage 60 (2023): 106657, 10.1016/j.est.2023.106657.

[20] X. Bin , M. Sheng , Y. Luo , and W. Que , “Heterostructures of MoO_3_ Nanobelts Assembled on Delaminated V4C3T MXene Nanosheets For Supercapacitors With Excellent Room/High Temperature Performance,” Electrochimica Acta 446 (2023): 142070, 10.1016/j.electacta.2023.142070.

[21] X. Bin , B. Kong , M. Sheng , and W. Que , “Green in Situ Fabrication of 3D Porous MoO_3_/Ti_3_C_2_T_x_ Aerogel Films for Enhanced Supercapacitor Performance without Organic Solvents,” Advanced Materials Technologies 10, no. 19 (2025): 00380, 10.1002/admt.202500380.

[22] H. Zhang , Z. Li , Z. Hou , et al., “Self‐Assembly of MOF on MXene Nanosheets and in‐Situ Conversion into Superior Nickel Phosphates/MXene Battery‐Type Electrode,” Chemical Engineering Journal 425 (2021): 130602, 10.1016/j.cej.2021.130602.

[23] L. Xu , W. Zhou , S. Chao , et al., “Advanced Oxygen‐Vacancy Ce‐Doped MoO_3_ Ultrathin Nanoflakes Anode Materials Used as Asymmetric Supercapacitors With Ultrahigh Energy Density,” Advanced Energy Materials 12, no. 16 (2022): 2200101, 10.1002/aenm.202200101.

[24] S. Liu , C. Xu , H. Yang , et al., “Atomic Modulation Triggering Improved Performance of MoO_3_ Nanobelts for Fiber‐Shaped Supercapacitors,” Small 16, no. 6 (2020): 1905778, 10.1002/smll.201905778. 31957981

[25] Y. Niu , X. Li , H. Su , J. Li , and Y. Qi , “Formation of Three Dimensional Porous h‐MoO_3_ Architecture and its Application in Supercapacitors,” Materials Letters 316 (2022): 132062, 10.1016/j.matlet.2022.132062.

[26] H. Zhang , X. Li , N. Zhang , et al., “Enhanced Hydrogen‐Ion Storage Performance of Molybdenum Trioxide Nanoribbons Doped by Oxygen Vacancies,” ACS Applied Materials & Interfaces 17, no. 17 (2025): 25684–25692, 10.1021/acsami.5c01903. 40238347

[27] X. Bin , M. Sheng , and W. Que , “Oxygen Vacancy‐Enriched MXene Nanosheets Coated With MoO_3–x_ for Glucose Monitoring,” ACS Applied Nano Materials 8, no. 18 (2025): 9356–9363, 10.1021/acsanm.5c00903.

[28] H.‐S. Kim , J. B. Cook , H. Lin , et al., “Oxygen Vacancies Enhance Pseudocapacitive Charge Storage Properties of MoO_3−x_ ,” Nature Materials 16, no. 4 (2017): 454–460, 10.1038/nmat4810. 27918566

[29] S. Sun , C. Liu , S. Zhang , et al., “Rich Oxygen Vacancies Mediated Metal‐Insulator Transition Materials toward Ultrasensitive Sensing and Energy Conversion,” Nano Energy 126 (2024): 109606, 10.1016/j.nanoen.2024.109606.

[30] C. Huang , W. Zhang , and W. Zheng , “The Debut and Spreading the Landscape for Excellent Vacancies‐Promoted Electrochemical Energy Storage of Nano‐Architected Molybdenum Oxides,” Materials Today Energy 30 (2022): 101154, 10.1016/j.mtener.2022.101154.

[31] J. Yang , X. Xiao , P. Chen , et al., “Creating Oxygen‐Vacancies in MoO_3_‐ Nanobelts Toward High Volumetric Energy‐Density Asymmetric Supercapacitors With Long Lifespan,” Nano Energy 58 (2019): 455–465, 10.1016/j.nanoen.2019.01.071.

[32] Y. Li , D. Wang , Q. An , B. Ren , Y. Rong , and Y. Yao , “Flexible Electrode For Long‐Life Rechargeable Sodium‐Ion Batteries: Effect of Oxygen Vacancy in MoO_3−x_ ,” Journal of Materials Chemistry A 4, no. 15 (2016): 5402–5405, 10.1039/C6TA01342B.

[33] T. Li , M. Beidaghi , X. Xiao , et al., “Ethanol Reduced Molybdenum Trioxide for Li‐Ion Capacitors,” Nano Energy 26 (2016): 100–107, 10.1016/j.nanoen.2016.05.004.

[34] H. Shu , M. Zhao , C. Tang , K. Ma , and X. Li , “Overturning Lactic Acid Hydrogenation Regioselectivity via Ru‐stabilized MoO_3‐x_ Overlayers,” Applied Surface Science 684 (2025): 161844, 10.1016/j.apsusc.2024.161844.

[35] Z. Li , D. Wu , W. Gong , et al., “Highly Efficient Photocatalytic CO_2_ Methanation over Ru‐Doped TiO_2_ with Tunable Oxygen Vacancies,” Chinese Journal of Structural Chemistry 41, no. 12 (2022): 2212043–2212050, 10.14102/j.cnki.0254-5861.2022-0212.

[36] M. Peng , D. Zhang , L. Zheng , et al., “Hierarchical Ru‐Doped Sodium Vanadium Fluorophosphates Hollow Microspheres as a Cathode of Enhanced Superior Rate Capability and Ultralong Stability for Sodium‐Ion Batteries,” Nano Energy 31 (2017): 64–73, 10.1016/j.nanoen.2016.11.023.

[37] Y. Zhang , P. Chen , Q. Wang , et al., “High‐Capacity and Kinetically Accelerated Lithium Storage in MoO_3_ Enabled by Oxygen Vacancies and Heterostructure,” Advanced Energy Materials 11, no. 31 (2021): 2101712, 10.1002/aenm.202101712.

[38] M. A. Camacho‐López , L. Escobar‐Alarcón , M. Picquart , R. Arroyo , G. Córdoba , and E. Haro‐Poniatowski , “Micro‐Raman Study of the M‐MoO_2_ to Α‐MoO_3_ Transformation Induced by Cw‐Laser Irradiation,” Optical Materials 33, no. 3 (2011): 480–484, 10.1016/j.optmat.2010.10.028.

[39] B. C. Windom , W. G. Sawyer , and D. W. Hahn , “A Raman Spectroscopic Study of MoS_2_ and MoO_3_: Applications to Tribological Systems,” Tribology Letters 42, no. 3 (2011): 301–310, 10.1007/s11249-011-9774-x.

[40] M. Dieterle and G. Mestl , “Raman Spectroscopy Of Molybdenum Oxides,” Physical Chemistry Chemical Physics 4, no. 5 (2002): 822–826, 10.1039/B107046K.

[41] X. Zheng , S. S. Mofarah , C. Cazorla , et al., “Decoupling the Impacts of Engineering Defects and Band Gap Alignment Mechanism on the Catalytic Performance of Holey 2D CeO_2−x_ ‐Based Heterojunctions,” Advanced Functional Materials 31, no. 38 (2021): 2103171, 10.1002/adfm.202103171.

[42] Y. Q. Chang , P. W. Wang , S. L. Ni , Y. Long , and X. D. Li , “Influence of Co Content on Raman and Photoluminescence Spectra of Co Doped ZnO Nanowires,” Journal of Materials Science & Technology 28, no. 4 (2012): 313–316, 10.1016/S1005-0302(12)60060-7.

[43] J. Kaur , J. Shah , R. K. Kotnala , and K. C. Verma , “Raman Spectra, Photoluminescence and Ferromagnetism of Pure, Co and Fe Doped SnO_2_ Nanoparticles,” Ceramics International 38, no. 7 (2012): 5563–5570, 10.1016/j.ceramint.2012.03.075.

[44] W. Li , Y. Zhao , Y. Liu , et al., “Exploiting Ru‐Induced Lattice Strain in CoRu Nanoalloys for Robust Bifunctional Hydrogen Production,” Angewandte Chemie International Edition 60, no. 6 (2021): 3290–3298, 10.1002/anie.202013985. 33105050

[45] L.‐Y. Kuo , C. Roitzheim , H. Valencia , et al., “Doping‐Induced Surface and Grain Boundary Effects in Ni‐Rich Layered Cathode Materials,” Small 20, no. 26 (2024): 2307678, 10.1002/smll.202307678. 38258588

[46] C. Li , H. Jang , M. G. Kim , L. Hou , X. Liu , and J. Cho , “Ru‐Incorporated Oxygen‐Vacancy‐Enriched MoO_2_ Electrocatalysts for Hydrogen Evolution Reaction,” Applied Catalysis B: Environment and Energy 307 (2022): 121204, 10.1016/j.apcatb.2022.121204.

[47] L. Li , S. Liu , C. Zhan , et al., “Surface and Lattice Engineered Ruthenium Superstructures towards High‐Performance Bifunctional Hydrogen Catalysis,” Energy & Environmental Science 16, no. 1 (2023): 157–166, 10.1039/D2EE02076A.

[48] W. Zhong , R. Zhao , Y. Zhu , Y. Xu , W. Chen , and C. Peng , “Vacancy Engineering on MnSe Cathode Enables High‐Rate and Stable Zinc‐Ion Storage,” Advanced Functional Materials 35, no. 17 (2025): 2419720, 10.1002/adfm.202419720.

[49] Q. Zhu , S. Jiang , K. Ye , et al., “Hydrogen‐Doping‐Induced Metal‐Like Ultrahigh Free‐Carrier Concentration in Metal‐Oxide Material for Giant and Tunable Plasmon Resonance,” Advanced Materials 32, no. 50 (2020): 2004059, 10.1002/adma.202004059. 33174328

[50] Y. Wang , X. Wang , X. Li , et al., “Intercalating Ultrathin MoO3 Nanobelts into MXene Film with Ultrahigh Volumetric Capacitance and Excellent Deformation for High‐Energy‐Density Devices,” Nano‐Micro Letters 12, no. 1 (2020): 115, 10.1007/s40820-020-00450-0. PMC7770681 34138117

[51] K. Zhou , W. Zhou , X. Liu , et al., “Ultrathin MoO_3_ Nanocrystals Self‐Assembled on Graphene Nanosheets via Oxygen Bonding as Supercapacitor Electrodes of High Capacitance and Long Cycle Life,” Nano Energy 12 (2015): 510–520, 10.1016/j.nanoen.2015.01.017.

[52] X. Li , X. Wang , M. Li , et al., “Built‐In Electric Field Enhancement Strategy Induced by Cross‐Dimensional Nano‐Heterointerface Design for Electromagnetic Wave Absorption,” Advanced Functional Materials 35, no. 18 (2025): 2407217, 10.1002/adfm.202407217.

[53] N. Zhao , H. Fan , M. Zhang , et al., “Simple Electrodeposition of MoO_3_ Film on Carbon Cloth for High‐Performance Aqueous Symmetric Supercapacitors,” Chemical Engineering Journal 390 (2020): 124477, 10.1016/j.cej.2020.124477.

[54] H. Ma , J. Liang , J. Qiu , et al., “A Biocompatible Supercapacitor Diode With Enhanced Rectification Capability Toward Ion/Electron‐Coupling Logic Operations,” Advanced Materials 35, no. 25 (2023): 2301218, 10.1002/adma.202301218. 36940232

[55] J. W. Gittins , K. Ge , C. J. Balhatchet , P.‐L. Taberna , P. Simon , and A. C. Forse , “Understanding Electrolyte Ion Size Effects on the Performance of Conducting Metal–Organic Framework Supercapacitors,” Journal of the American Chemical Society 146, no. 18 (2024): 12473–12484, 10.1021/jacs.4c00508. PMC11082900 38716517

[56] B. Pal , S. Yang , S. Ramesh , V. Thangadurai , and R. Jose , “Electrolyte Selection for Supercapacitive Devices: A Critical Review,” Nanoscale Advances 1, no. 10 (2019): 3807–3835, 10.1039/C9NA00374F. PMC9417677 36132093

[57] R. Wang , Q. Li , L. Cheng , et al., “Electrochemical Properties of Manganese Ferrite‐Based Supercapacitors in Aqueous Electrolyte: The Effect of Ionic Radius,” Colloids and Surfaces A: Physicochemical and Engineering Aspects 457 (2014): 94–99, 10.1016/j.colsurfa.2014.05.059.

[58] V. Augustyn , P. Simon , and B. Dunn , “Pseudocapacitive Oxide Materials for High‐Rate Electrochemical Energy Storage,” Energy & Environmental Science 7, no. 5 (2014): 1597–1614, 10.1039/C3EE44164D.

[59] C. Costentin and J.‐M. Savéant , “Energy Storage: Pseudocapacitance in Prospect,” Chemical Science 10, no. 22 (2019): 5656–5666, 10.1039/C9SC01662G. PMC6563784 31293750

[60] P. Simon and Y. Gogotsi , “Materials for Electrochemical Capacitors,” Nature Materials 7, no. 11 (2008): 845–854, 10.1038/nmat2297. 18956000

[61] M. Okoshi , Y. Yamada , S. Komaba , A. Yamada , and H. Nakai , “Theoretical Analysis of Interactions between Potassium Ions and Organic Electrolyte Solvents: A Comparison with Lithium, Sodium, and Magnesium Ions,” Journal of the Electrochemical Society 164, no. 2 (2017): A54–A60, 10.1149/2.0211702jes.

