# Supplementary material for: Ru‐Doping‐Engineered Oxygen Vacancies in MoO3‐ x Nanostructured Films With Ultrahigh Capacitance for Flexible Asymmetric Supercapacitors
Source: Adv Sci (Weinh). 2026 Aug 17:e77131. Online ahead of print. doi: 10.1002/advs.77131 (PMC13479587; doi:10.1002/advs.77131)
Supplement: Supplementary file 1 [file ADVS-9999-e77131-s001.docx]

**Supporting Information**

**Ru-Doping-Engineered Oxygen Vacancies in MoO_3-_*_x_* Nanostructured Films with Ultrahigh Capacitance for** **Flexible Asymmetric Supercapacitors**

**Xiaoqing Bin ^a, b^, Minhao Sheng ^a, b^, Norman C.-R. Chen ^b, c, d^, Xiangyang Liu** **^b^, Yingji Zhao ^b^, Yingyi Liao ^a^, Wenxiu Que ^a, *^, Yusuke Asakura ^b, *^, Yusuke Yamauchi ^b, e, f *^**

^a^ Electronic Materials Research Laboratory, Key Laboratory of the Ministry of Education, International Center for Dielectric Research, Shaanxi Engineering Research Center of Advanced Energy Materials and Devices, School of Electronic Science and Engineering, Xi’an Jiaotong University, Xi’an 710049, P. R. China

^b^ Department of Materials Process Engineering, Graduate School of Engineering, Nagoya University, Nagoya 464-8603, Japan

^c^ Molecular Science and Technology Program, Taiwan International Graduate Program, Academia Sinica, Taipei, 10617 Taiwan

^d^ International Graduate Program of Molecular Science and Technology (NTU-MST), National Taiwan University, Taipei, 10617 Taiwan

^e^ Australian Institute for Bioengineering and Nanotechnology (AIBN), The University of Queensland, Brisbane, Queensland 4072, Australia

^f^ Department of Convergent Biotechnology and Advanced Materials Science, Kyung Hee University, 1732, Deogyeong-daero, Giheung-gu, Yongin-si, Gyeonggi-do 17104, South Korea

* wxque@mail.xjtu.edu.cn; asa.y@nagoya-u.jp; y.yamauchi@uq.edu.au

**Experimental Section**

**Electrode preparation**

**Materials:** Molybdenum powder (Mo, 99.0% metals basis) and ruthenium (III) chloride (RuCl_3_) were purchased from Shanghai Aladdin Biochemical Technology Co., Ltd. Hydrogen peroxide (H_2_O_2_, 25-35 wt% in H_2_O) was from Shanghai Macklin Biochemical Co., Ltd., China. All chemical reagents were directly used as purchased without further purification. Moreover, ultrapure (UP) water (18.25 MΩ· cm) was used throughout the study.

**Synthesis of** **oxygen-vacancy MoO_3_ and Ru-doped MoO_3-_*_x_* flexible film electrodes:**

(1) Preparation of the precursor solution: Under ice-bath conditions, 0.7 g of molybdenum (Mo) powder was slowly added to 10 mL of a hydrogen peroxide (H_2_O_2_) solution (25-35 wt%) under continuous stirring. The reaction was allowed to proceed until a clear, pale-yellow solution was obtained. Subsequently, 50 mL of deionized water and 10 mL of ethanol were introduced into the solution, and the mixture was stirred at approximately 200 rpm until homogeneous.

(2) Doping with Ruthenium: While maintaining continuous stirring, different amounts of ruthenium (III) chloride (RuCl_3_), specifically 0 mg, 1 mg, 3 mg, and 6 mg, were slowly added to the respective precursor solutions. The corresponding samples were designated as MoO_3-_*_x_*, Ru-MoO_3-_*_x_*-1, Ru-MoO_3-_*_x_*-3, and Ru-MoO_3-_*_x_*-6, respectively. Vigorous effervescence (bubbling) was observed upon the addition of RuCl_3_. The reaction was allowed to proceed to completion with a total stirring time of approximately 2 h.

(3) Solvothermal Reaction: The resulting solutions were then transferred into a 100 mL Teflon-lined stainless-steel autoclave. The autoclave was sealed and heated in a drying oven at 140 °C for 18 h at a heating rate of 5 ℃ min^-1^.

(4) Post-Solvothermal Processing: After the autoclave was allowed to cool naturally to room temperature, the resulting reaction suspension was collected. The mixture was transferred to several 50 mL centrifuge tubes, shaken for 5 min, and then subjected to ultrasonication for 10 min at a power of 100-200 W to ensure uniform dispersion. The dispersed solution was then centrifuged at 3500 rpm for 1 min per cycle. During the initial two centrifugation cycles, the supernatant was discarded, and the precipitate was redispersed in ultrapure water for washing. From the third centrifugation cycle onward, the supernatant was collected. This centrifugation-collection process was repeated multiple times to obtain concentrated suspensions of the oxygen-vacancy MoO_3-_*_x_*, and the ruthenium-doped oxygen-vacancy MoO_3-_*_x_* nanomaterial with a final concentration of approximately 3 mg mL^-1^.

(5) Formation of the Flexible Film Electrode: The as-prepared suspension (approximately 3 mg mL^-1^) was subjected to vacuum filtration using a mixed cellulose ester (MCE) membrane (pore size: 0.22 μm, diameter: 47 mm) to form a uniform freestanding film. By controlling the volume of the suspension (about 40 mL), the mass loading of the film electrodes was maintained at 5.24 mg cm^-2^, 5.68 mg cm^-2^, 4.80 mg cm^-2^, and 5.24 mg cm^-2^ for MoO_3-_*_x_*, Ru-MoO_3-_*_x_* -1, Ru-MoO_3-_*_x_* -3, Ru-MoO_3-_*_x_* -6, respectively. The resulting films were then dried under vacuum at 35 °C for 24 h, and carefully peeled off from the membrane. To prepare the working electrodes, circular discs with a diameter of 5.4 mm were punched from the freestanding films. These electrodes were tested directly without additional substrates, serving as both the active material and the current collector, thereby demonstrating their self-supported nature.

**Preparation of the counter electrode:** First, a pre-mixed slurry was prepared by combining 95 wt% activated carbon (YP-50F), and 5 wt% polytetrafluoroethylene (PTFE) binder (50 wt% in ethanol). The resulting slurry was rolled into a flat film using a glass cylinder. The film was subsequently dried in a vacuum oven at 60 °C for 12 h. Finally, circular activated carbon electrodes with a diameter of 5.4 mm were obtained using a hole punch and used as the counter electrodes. The mass loading of the counter electrodes was maintained at approximately 8.73 mg cm^-2^.

**Preparation of the** **positive electrode:** First, the active material slurry was prepared by mixing 95 wt% activated carbon (YP-50F) and 5 wt% polyvinylidene fluoride binder (PVDF) in *N*-methyl-2-pyrrolidone (NMP) as the solvent. The resulting mixture was stirred to form a homogeneous slurry and uniformly coated onto carbon cloth (Φ = 5.4 mm) to fabricate the positive electrode. The mass loading of the positive electrodes was about 6.98 mg cm^-2^. The coated electrodes were finally dried in a vacuum oven at 60°C for 12 h.

**Preparation for the** **PVA-H_2_SO_4_ hydrogel electrolyte:** 4 g of PVA was dissolved in a mixture of 28 mL of 3 M H_2_SO_4_ solution and 12 mL of ultrapure water at 90 ℃ under continuous stirring for approximately 60 min. After cooling to room temperature and degassing, the solution was cast into glass Petri dishes and subjected to two freeze-thaw cycles (freezing at -20 °C for 6 h and thawing at room temperature for 6 h per cycle) to yield the physically cross-linked hydrogel.

**Preparation** **for the 1 M EMIMTFSI/LiTFSI/ACN organic electrolyte**: 2.56 mL of EMIMTFSI and 2.87 g of LiTFSI (molar ratio of EMIMTFSI: LiTFSI = 1:1) were dissolved in 7.44 mL of acetonitrile (ACN), and the mixture was stirred for 2 h. **Material characterization:** The X-ray diffraction (XRD) patterns of the as-prepared samples were measured using a Rigaku D/max 2200pc diffractometer with Cu Kα radiation (λ = 1.5418 Å) in a 2*θ* angular range of 3-80°. The morphological properties of the samples were observed by Aberration-Corrected Scanning Transmission Electron Microscopy (AC-STEM, JEM-ARM200F) coupled with elemental mapping. The elemental compositions and valence states of the samples were characterized by X-ray photoelectron spectroscopy (XPS, VG Thermo ESCALAB 250 spectrometer) using Al Kα X-ray radiation. The X-ray absorption fine structure (XAFS) measurements were performed at the TPS44A beamline at the NSRRC in Taiwan. Ultraviolet Photoelectron Spectroscopy (UPS) measurements were performed using a VG ESCA Lab system under ultrahigh vacuum (UHV) conditions. The analysis utilized a He I excitation source (21.22 eV). A sample bias of -5.0 V was applied to determine the secondary electron cutoff (SECO). The energy scale was calibrated by referencing the Fermi edge (E_f_ = 0 eV) of a clean polycrystalline Ag foil. Ultraviolet–visible (UV–vis) absorption spectra were recorded using a Lambda 950 UV–vis–near-infrared spectrophotometer (PerkinElmer). Inductively coupled plasma mass spectrometry (ICP–MS) measurements were performed using a NexION 350D ICP–MS system (PerkinElmer). Electron paramagnetic resonance (EPR) spectra were recorded using a JES-FA200 spectrometer (JEOL Ltd.). Raman spectra were collected using a inVia Qontor Raman microscope (Renishaw).

**Computational details:** All the calculations were carried out using the Vienna Ab initio Simulation Package (VASP) with the frozen-core all-electron projector-augment-wave (PAW) method.^1, 2^ The generalized gradient approximation (GGA) proposed by Perdew-Burke-Ernzerhof (PBE) was selected for the exchange-correlation potential.^3^ The electronic band structures and density of states (DOS) were subsequently calculated based on the optimized structures. The cut-off energy for the plane wave was set to 520 eV. The Brillouin-zone integration was performed using Γ-centered k-point mesh in the three-dimensional Brillouin zone. The thresholds were 10^-5^ eV and 0.02 eV/Å for electronic and ionic relaxations, respectively.

**Electrochemical measurements:** The electrochemical measurements were performed by a CHI 660E electrochemical workstation (CH Instruments, China) at room temperature. In the three-electrode system, 3 M aqueous H_2_SO_4_ was chosen as the electrolyte, the MoO_3-_*_x_* and Ru-MoO_3-_*_x_* flexible films with varying Ru concentrations electrodes (Φ 5.4 mm in diameter), the activated carbon film (Φ 5.4 mm in diameter), and Hg/Hg_2_SO_4_ (sat. K_2_SO_4_) electrode were used as the working electrodes, the counter electrode, and the reference electrode, respectively. The electrochemical performance of the film electrodes was investigated by a series of electrochemical measurements. Over the potential range from -0.95 V to -0.15 V, the cyclic voltammetry (CV) measurement was performed at different scan rates (2, 5, 10, 20, 50, 100, and 200 mV s^-1^), the galvanostatic charge-discharge (GCD) measurement was conducted at various current densities (1, 2, 3, 4, 5, and 10 A g^-1^). Moreover, the electrochemical impedance spectroscopy (EIS) was measured over a frequency range of 0.01 Hz to 100 kHz. The long-term cycling stability test was evaluated using a LANHE CT2001A tester (LAND, China), repeating the GCD test for 5000 cycles at 2 A g^-1^.

Gravimetric capacitance (*C*_g1_, F g^-1^) of the working electrode in the three-electrode system was calculated from the CV curves using the following formula:

*C*_g1_ *=* ʃ *I*_1_ d*V /* (2 *m s* Δ*V*)

where *I*_1_ (A) is the measured current response of the CV curve, *s* (V s^-1^) is the scan rate, Δ*V* (V) is the potential window, and *m* (g) is the mass of the working electrode (the film or active material).

The gravimetric capacitance (*C*_g2_, F g^-1^) of the working electrode in the three-electrode system calculated from the GCD curves is given by the following formula:

*C*_g2_ *=* *I*_2_ Δ*t / (m* Δ*V )*

where *I*_2_ (A) is the constant discharging current of the GCD curve, Δ*t* is the discharging time of the GCD curve, Δ*V* (V) is the potential window, and *m* (g) is the mass of the working electrode (the film or active material).

The electrochemical performance of supercapacitor devices was evaluated in three different electrolytes: 3 M H_2_SO_4_, PVA-H_2_SO_4_ hydrogel, and 1 M EmimTFSI/LiTFSI in acetonitrile (ACN). The asymmetric supercapacitor (ASC) was assembled by using AC as the positive electrode and the optimal Ru-MoO_3_*_-x_*-3 flexible film as the negative electrode. The electrochemical testing conditions were the same as those of the three-electrode system. For the ASC, the voltage window was set from 0 to 1.6 V in the acidic electrolyte (H_2_SO_4_ and PVA-H_2_SO_4_), while it was extended to 2.4 V in the organic electrolyte (EmimTFSI/LiTFSI). In addition, prior to the assembly of the Ru-MoO_3_*_-x_*-3 // AC ASC, the mass loading of the positive and negative electrodes was balanced based on the following formula:

*m*_+_/*m*_-_=C_s-_ Δ*V*_-_ /C_s+_ Δ*V*_+_

where m_+_ and m_-_ are the masses of the film or active material (g), C_s+_ and C_s-_ are the specific capacitances (F g^-1^), Δ*V*_+_ and Δ*V*_-_ are the potential windows (V) of the positive and negative electrodes, respectively.

Gravimetric capacitance, *C*_g_*, _c_* (F g^-1^), of the supercapacitors were calculated by the following formula:

*C*_g_,_c_ = *I*_3_ Δ*t*_0_ */ (m_c_* Δ*V*_0_ *)*,

where *m*_c_ (g) is the total mass of electrodes for the supercapacitors, *I*_3_ (A) is the constant discharging current of the GCD curve, Δ*t*_0_ is the discharging time of the GCD curve, Δ*V*_0_ (V) is the voltage range for the device.

Energy density (*E*, Wh kg^-1^) and power density (*P*, W kg^-1^) of the asymmetric supercapacitor can be calculated via the following equations:

*E* = *C*_g_,_c_ (Δ*V*_0_)^2^ /7.2

*P* = 3*600 E/Δt*_0_,

**Calculation**

The electrical conductivity of the film electrodes was measured at room temperature by using a four-point probe. The square resistance values were measured by using the B2912A Precision Source/Measure Unit. The four-point probe method depends on a linear array of four equally spaced tips that were pressed on the surface of the sample. Thus, the resistivity was calculated by the following equation:^4^

ρ_0_ = BtR	(S1)

where ρ_0_ is resistivity (Ω cm), t is thickness of the film sample (cm), B is the correction factor, R is the resistance measured directly using the four-point probe ((R=V/I), Ω), as presented in Table S1. In the limit, when the spacing of probe tips is much less than the lateral dimension of the sample, B becomes (π/ln2) = 4.53. Thus, the electrical conductivity can be obtained by the following equation:

σ = 1/ρ_0_ (S2)

where σ is electrical conductivity (S cm^-1^).

**Electrochemical kinetic** **equations:**

*i**_peak current_* = *i*_cap_ + *i*_diff_ = *av^b^*	(S3)

log *i_peak current_* = log *a* + *b* log*v*	(S4)

where the *i_peak current_* was measured in the CV test at different scan rates (*v*, mV s^-1^) from 2 mV s^-1^ to 20 mV s^-1^. The current (*i*) consists of two parts, including the diffusion-controlled process (*i*_diff_) and the surface-capacitance-dominated process (*i*_cap_). The values "a" and "b" are the fitting parameters. Typically, if the b value is close to 0.5, the process is diffusion-controlled. If the b value approaches 1.0, it is the surface capacitance dominated process. A b value between 0.5 and 1.0 indicates that the charge-storage process involves both diffusion-controlled battery-type behavior and surface-controlled capacitive behavior.

*i* (V) = *i*_cap_ + *i*_diff_ = *k*_1_*v* + *k*_2_*v*^1/2^	(S5)

*i* (V) / *v*^1/2^ = *k*_1_*v*^1/2^ + *k*_2_	(S6)

where the *k*_1_*v* represents the current from surface capacitive contribution, and the *k*_2_ *v*^1/2^ represents the current from diffusion contribution.

**Table S1**

Thickness, four-point-probe resistance, and electrical conductivity of the film electrodes

| Sample | Thickness (cm) | Resistance measured by the four-point probe (Ω) | Electrical conductivity (10^-3^ S cm^-1^) |
| --- | --- | --- | --- |
| MoO_3-_*_x_*  Ru-MoO_3-_*_x_*-1  Ru-MoO_3-_*_x_*-3  Ru-MoO_3-_*_x_*-6 | 0.0299  0.0280  0.0223  0.0249 | 3885.93  1514.47  1225.30  1362.68 | 1.90  5.21  8.08  6.51 |

**Table S2**

Peak-fit parameters of the high-resolution O 1s XPS spectrum for the MoO_3-_*_x_*

| **Peak** | **Position** | **Area** | **FWHM** |
| --- | --- | --- | --- |
| **O_L_** | **530.6** | **134649.3** | **1.38** |
| **O_V_** | **532.1** | **8926.293** | **1.56** |

**Table S3**

Peak-fit parameters of the high-resolution O 1s XPS spectrum for the Ru-MoO_3-_*_x_*-1

| **Peak** | **Position** | **Area** | **FWHM** |
| --- | --- | --- | --- |
| **O_L_** | **530.6** | **111249.4** | **1.23** |
| **O_V_** | **532.1** | **10176.72** | **1.56** |

**Table S4**

Peak-fit parameters of the high-resolution O 1s XPS spectrum for the Ru-MoO_3-_*_x_*-3

| **Peak** | **Position** | **Area** | **FWHM** |
| --- | --- | --- | --- |
| **O_L_** | **530.7** | **117625.0** | **1.22** |
| **O_V_** | **532.1** | **21795.63** | **1.51** |

**Table S5**

Peak-fit parameters of the high-resolution O 1s XPS spectrum for the Ru-MoO_3-_*_x_*-6

| **Peak** | **Position** | **Area** | **FWHM** |
| --- | --- | --- | --- |
| **O_L_** | **530.6** | **109469.9** | **1.34** |
| **O_V_** | **532.1** | **13155.08** | **1.54** |

**Figure S1.** (a) STEM image and the **corresponding elemental distribution mapping of (b) Mo** element, and (c) O element of MoO_3-_*_x_*.

**Figure S2.** (a) WT-EXAFS spectra and (b) corresponding 3D images of Ref. Mo.

**Figure S3**. Quantitative correlation analysis of oxygen vacancies in MoO_3-_*_x_* and Ru-MoO_3-_*_x_* samples. The scatter plot displays the correlation between the EPR signal intensity, which reflects oxygen-vacancy-related unpaired-electron species, and the surface oxygen-vacancy content estimated by the O_v_ area ratio in the XPS O 1s spectra. The solid line represents the linear fit of the experimental data points, yielding a high Pearson correlation coefficient of r = 0.88, indicating apositive correlation between two independently measured defect-related signals.

**Figure S4.** Tauc plots of (a) MoO_3-_*_x_*, (b) Ru-MoO_3-_*_x_*-1, (c) Ru-MoO_3-_*_x_*-3, and (d) Ru-MoO_3-_*_x_*-6 films.

**Figure S5.** (a) DFT-optimized atomic configurations of MoO_3-_*_x_* and Ru-MoO_3-_*_x_*. Partial density of states (PDOS) of (b, c) Mo and O in MoO_3-_*_x_*, and (d, e) Mo and O in Ru-MoO_3-_*_x_*.

**Figure S6.** Cycling stability and coulombic efficiency of the Ru-MoO_3-_*_x_*-3 film electrode.

**Figure S7.** CV and GCD curves of (a-b) Ru-MoO_3-_*_x_*-3//AC device in 3M H_2_SO_4_ electrolyte, (c-d) Ru-MoO_3-_*_x_*-3//AC device in PVA-H_2_SO_4_ electrolyte, and (e-f) Ru-MoO_3-_*_x_*-3//AC device in 1 M EmimTFSI/LiTFSI/ACN electrolyte.

**Figure S8.** Cycling stability and coulombic efficiency of the Ru-MoO_3-_*_x_*-3 //AC device in 3M H_2_SO_4_ electrolyte.

**Figure S9.** CV and GCD curves of (a-b) MoO_3-_*_x_*, (c-d) Ru-MoO_3-_*_x_*-1, (e-f) Ru-MoO_3-_*_x_*-3, and (g-h) Ru-MoO_3-_*_x_*-6 film electrodes.

**References**

1.	Kresse, G.; Joubert, D., From ultrasoft pseudopotentials to the projector augmented-wave method. *Physical Review B* **1999,** *59*, 1758-1775.

2.	Kresse, G.; Furthmüller, J., Efficient iterative schemes for ab initio total-energy calculations using a plane-wave basis set. *Physical Review B* **1996,** *54*, 11169-11186.

3.	Perdew, J. P.; Burke, K.; Ernzerhof, M., Generalized gradient approximation made simple. *Physical Review Letters* **1996,** *77*, 3865-3868.

4.	Luo, Y. Y.; Yang, C. H.; Tian, Y. P.; Tang, Y.; Yin, X. T.; Que, W. X., A Long Cycle Life Asymmetric Supercapacitor Based on Advanced Nickel-Sulfide/Titanium Carbide (MXene) Nanohybrid and MXene Electrodes *. J. Power Sources* 2020, **450**, 227694.
